# Heat Capacity Estimation Using a Complete Set of Homodesmotic Reactions for Organic Compounds

**DOI:** 10.3390/molecules27227814

**Published:** 2022-11-13

**Authors:** Sergey L. Khursan

**Affiliations:** Ufa Institute of Chemistry of Ufa Federal Research Center of Russian Academy of Sciences, 71 Prospect Oktyabrya, 450054 Ufa, Russia; khursansl@anrb.ru; Tel.: +7-(347)-2356066

**Keywords:** homodesmotic reactions, isobaric molar heat capacity, complete set of HDRs, group separation reactions

## Abstract

Reliable information about isobaric heat capacities *C_P_* is necessary to determine the energies of organic compounds and chemical processes at an arbitrary temperature. In this work, the possibility of theoretical estimation of *C_P_* by the homodesmotic method is analyzed. Three cases of *C_P_* calculation applying the methodology of the complete set of homodesmotic reactions (CS HDRs) are considered: the gas- and liquid-phase *C_P_* of organic compounds of various classes at 298 K (the mean absolute value of reaction heat capacity, MA Δ*C_P_* = 1.44 and 2.83 J/mol·K for the gas and liquid phase, correspondingly); and the gas-phase *C_P_* of *n*-alkanes C_2_–C_10_ in the temperature range of 200–1500 K with an average error in calculating the heat capacity of 0.93 J/mol·K. In the latter case, the coefficients of the Shomate equation are determined for all *n*-alkanes that satisfy the homodesmoticity condition. New values of gas- and liquid-phase heat capacities are obtained for 41 compounds. The CS HDRs-based approach for estimating the *C_P_* of organic compounds is characterized by high accuracy, which is not inferior to that of the best *C_P_*-additive schemes and allows us to analyze the reproducibility of the calculation results and eliminate unreliable reference data.

## 1. Introduction

The prediction of the organic compound reactivity based on the structural data of a molecule is one of the fundamental problems of physical organic chemistry. Significant progress in this direction has been achieved with the development of additive [1,2] and comparative methods [3,4,5] for the a priori estimation of the thermodynamic constants of organic compounds. In combination with modern computational procedures of quantum chemistry, the methods of thermochemical calculations based on the principle of maximal similarity of the studied compound with a number of auxiliary reference structures have come to the fore. To determine the energy of chemical compounds (enthalpy of formation Δ*_f_H*°, bond strength *D*, etc.), it has been proposed to use homodesmotic reactions (HDRs) [4,5,6,7], which are formal thermochemical transformations constructed to keep several balances on both sides of the equation: material, isogyric, bond, group, as well as the balance of non-valence effects. The common effect of these balances is the energy balance of the formal reaction, i.e., the fulfillment of the thermoneutrality condition of HDR (Δ*_r_H*° = 0) [8]. Homodesmotic methodology has shown its effectiveness in solving various problems of physical organic chemistry and has entered the research arsenal of a large number of scientific groups around the world. Thus, in the 21st century, more than 500 scientific materials have been published that use the concept of homodesmoticity to obtain various types of thermochemical information. It may provide few illustrative examples of recent publications using the homodesmotic method for calculating the enthalpy characteristics of organic molecules and radicals [8,9,10,11,12,13,14,15,16], quantitative description of aromaticity [17,18,19], determination of strain energies in cyclic structures and compounds of a complex structure [20,21,22,23,24,25,26,27]. As with additive approaches, the homodesmotic method is convenient for the algorithmization and application of computational technologies in thermochemical analysis [28,29,30,31,32,33].

As a rule, the thermochemical characteristics of organic molecules are calculated for *T* = 298.15 K. This makes it difficult to predict the reactivity for different temperatures. To solve this problem, several methods have been developed for determining the heat capacity of a substance (*C_P_* or *C_V_*). Theoretical calculations of heat capacity can be carried out using the conceptual apparatus of statistical mechanics and molecular thermodynamics [34] or the semi-empirical schemes based on the principle of additivity (see the brief anniversary review of W.E. Acree and J.D. Chickos) [35]. Obviously, estimates of *C_P_* are also possible using the principle of similarity in a comparative calculation, in particular, using the method of homodesmotic reactions. However, to the best of our knowledge, this possibility has not yet been explored.

An arbitrarily chosen HDR is often used in homodesmotic calculations. It is obvious that more reliable results can be obtained by the multiple evaluation of the thermochemical quantity. In our recent works [8,30,31], we proposed the construction of a complete set of HDRs for the compound under study (CS HDRs) using graph theory. The main advantage of this approach is the estimation of studied quantity from several independent HDRs, which allows on this basis to control the reproducibility of the calculation, to eliminate doubtful results and, therefore, to increase the reliability of theoretical estimate. In the present work, the possibility of applying a homodesmotic method for calculating the gas-phase and liquid-phase heat capacities of organic compounds is tested, as well as the estimation of isobaric molar heat capacity using CS HDR formalism for the compounds of representative test set is performed.

## 2. Computational Details

A complete set of HDRs was constructed within the framework of the graph theory approach described in [30]. A studied compound is represented as a graph, where its vertices are internal or terminal thermochemical groups of atoms with only the first valence environment of the central atom. Thus, graph nodes correspond to single chemical bonds between groups. This graph can be associated with the group adjacency matrix, where units correspond to a chemical bond and zeros to its absence. A CS HDR for an arbitrary compound is constructed by zeroing out one or more elements of the adjacency matrix, which corresponds to breaking bonds between thermochemical groups. Combinatorial zeroing achieves 2^(*n*−1)^ adjacency matrix transformations and, accordingly, 2^(*n*−1)^ homodesmotic reactions in the complete set, *n* is the number of internal thermochemical groups in a molecule. The number of HDRs in the complete set can be smaller if the molecule has a symmetrical structure. The decomposition of the adjacency matrix generates new matrices corresponding to HDR products. Coreagents can be easily chosen according to the type of thermochemical groups between which the bond is broken. This procedure is facilely algorithmized [31] and can be used for automatically constructing the complete sets of HDRs for the analyzed structures with excluding linearly dependent homodesmotic reactions.

The quantum−chemical calculations were performed using Gaussian-09 software, revision C1 [36]. Three levels of theory were used throughout this work: B3LYP/6-31G(d)–Becke 3-parameter, Lee–Yang–Parr exchange–correlation functional combined with economical Pople basis set of double valence-splitting augmented by the set of d-polarization functions [37,38,39], M06-2X/cc-pVTZ–Minnesota 06 global hybrid functional for main group thermochemistry in combination with Dunning triple-zeta correlation-consistent basis set [40,41] and composite G4 method [42]. The treatment of any molecular structure includes the full optimization of geometric parameters, calculation of frequencies and thermochemical quantities: absolute enthalpy, free Gibbs energy, entropy and heat capacity at 298 K. No solvent effect was considered.

## 3. Results and Discussion

### 3.1. Terminology

Various formal processes designed according to certain rules are widely used in thermochemical calculations. They are, for example, the Born–Haber cycle or thermochemical cycles based on Hess’s law. Pople et al. introduced into practice new formal reactions based on several balances (isodesmic reactions) in relation to the determination of enthalpies of formation of organic compounds using quantum chemical (QC) methods [3]. The five-level hierarchy of these processes was further outlined by Wheeler [6,7] ranging from so-called isogyric reactions (balance in lone electron pairs and unpaired electrons) to hyperhomodesmotic reactions (balance in the number of atoms of a certain hybridization and bonds between these atoms). Each higher level of formal processes includes lower levels as substructural elements. The key carrier of energy in this representation is chemical bond (“desmos” in Greek), which explains the terminology. With all the internal logic of the proposed classification, it can hardly be applied in this work, since it operates with hydrocarbon compounds only. Therefore, we adhere below to a different terminology, in which the main carrier of energy is a group of atoms. This approach is widely practiced in additive schemes, so it is enough to mention the well-known group-additive method of Benson [2] in application to gas-phase Δ*_f_H*°, *S*°, and the *C_P_* estimation or the method of Zabransky and Ruzicka for the calculation of the liquid-phase molar heat capacities of organic compounds [43,44].

**Figure 1:**
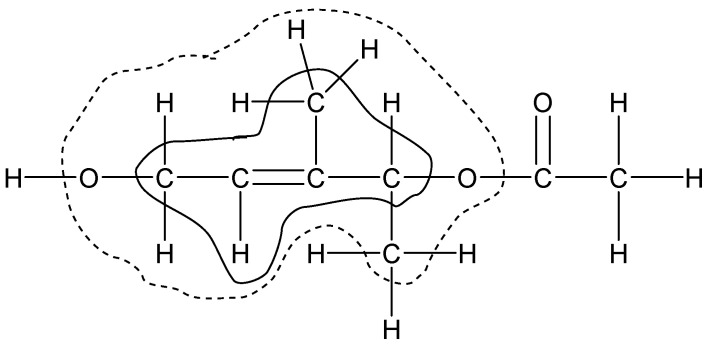
Thermochemical groups with a central “complex atom” C=C used in homodesmotic reactions of first level ([C=C-(C)(H);(C)_2_], inner area) and second level ([C=C-(C-(H)_2_(O))(H);(C-(H)_3_)(C-(C)(H)(O))], dotted outer area).

The second class of reference reactions in the Wheeler’s hierarchy, isodesmic, can be constructed as bond separation reactions. By analogy, at a higher level of similarity, a group separation reaction can be created. In other words, a homodesmotic process can be characterized as an isodesmic one with group balance. However, the term ‘thermochemical group’ can be interpreted in different ways. The group as it is understood in Benson’s method is not always possible to use in homodesmotic reactions, particularly, in the case of unsaturated compounds. Therefore, any multiple bond, including a carbon–carbon double or triple bond, is considered as an inseparable element, i.e., “complex atom”. In our representation, the smallest possible group includes an ordinary or complex central atom with a valence of ≥2 and ligands, i.e., atoms of the nearest (valence) environment. If all ligands except one are monovalent, then it corresponds to a terminal group, otherwise we have an internal group. We call this a homodesmotic reaction with a balance of smallest groups as HDR of the first level (HDR1). Accordingly, the homodesmotic reaction of level 2 (HDR2) deals with groups that include atoms of the first and second valence environments of the central atom, i.e., ligands of ligands. The further expansion of the group corresponds to HDR3 and so on. In the limit, the highest possible level of HDR for a compound containing *n* thermochemical groups includes a co-reagent with (*n* − 2) groups and two products with (*n* − 1) thermochemical groups each. For large molecules, this approach is often referred to as macroincrementing. The composition of thermochemical groups in the classification used is illustrated in Figure 1 using an arbitrary organic compound as an example.

From a practical point of view, an atom bonded to monovalent atoms can be considered as “complex atom” since the group separation reaction makes sense for internal (non-terminal) groups only. With this addition, the group of first level shown on Figure 1 has the composition [CH=C-(CH_2_);(CH)(CH_3_)], while that of second level is [CH=C-(CH_2_OH);(CH_3_)(CH(CH_3_)-(O))]. Obviously, the proposed hierarchy of homodesmotic reactions can be applied to any class of organic compounds with at least two internal thermochemical groups of any composition. The higher the level of HDR, the more strictly the principle of maximum similarity of reagents and products is fulfilled, and the more grounds for obtaining a correct prediction of organic compound properties. We took this circumstance into account when analyzing the heat capacity data.

The presence of all balances in a homodesmotic reaction ensures the thermoneutrality of the formal process, Δ*_r_H*° = 0. With this conclusion, the temperature is ignored, i.e., it may be tentatively assumed that the thermoneutrality condition for HDR is satisfied at any temperature, or, at least, in a certain temperature range. It can be concluded a priori that the deviation of Δ*_r_H*° from the “ideal” zero value is caused by the difference in the environment of the atoms, whose equivalence is assumed during the choice of reference reaction. Since a group balance takes place in HDRs, the first valence environment of all atoms on the left side of HDR1 has an analogue on the right side. The differences start from the second environment, i.e., from the environment of ligands. This effect should be small; moreover, the non-equivalence of the second environment may be overcome in HDR2 or higher levels. So, we can expect the condition Δ*_r_H*°(*T*) = 0 to be satisfied in a rather wide temperature interval. Since Δ*C_P_* = ∂(Δ*_r_H*°)/∂*T*, then the change in isobaric heat capacity is zero, Δ*C_P_* = 0 for the thermoneutral or temperature-independent HDR. This makes it possible to estimate *C_P_* for organic compound using known values of heat capacity of other HDR participants. Reliable information on heat capacities is scarcer than that for enthalpies of formation, so the use of complete sets of homodesmotic reactions for calculating *C_P_* is of great importance, since it allows to control the reproducibility of the results and reasonably filter out unreliable experimental data. Several simple but illustrative examples of the use of the CS HDR methodology for determining the isobaric molar heat capacity of organic compounds are considered below.

### 3.2. Isobaric Molar Heat Capacities at 298 K, the KIAS16 Test Set of Organic Compounds

The applicability of the homodesmotic method for evaluating *C_P_* of organic compounds was checked using the test set of 53 aliphatic organic compounds, including alkanes, alkenes, alkynes, alcohols, ethers, aldehydes, ketones and esters, as well as N-containing compounds: amines, amides and nitriles [8]. Together with the reference compounds, the KIAS16 test set deals with 80 CHNO-containing substances with the number of carbon atoms from one to six. The high accuracy of the homodesmotic calculation of gas-phase enthalpies of formation Δ*_f_H*°_298_ is demonstrated using from one to seven HDRs for each compound of the KIAS16 test set [8]. It was found that the thermal effect of HDRs is close to zero in most cases. This circumstance determined the choice of KIAS16 to test the possibility of reliable calculation of *C_P_* by the homodesmotic method. The *C_P_* experimental values for the gas and liquid phases were taken from the databases [45,46,47]. If *C_P_* at 298 K for a tested compound was determined in several works, then the average value was obtained by routine statistical treatment and removing doubtful measurements. The used *C_P_* values are shown in Supplement Materials, Table S1, as well as the isobaric molar heat capacities calculated at the B3LYP/6-31G(d) and M06-2X/cc-pVTZ levels of theory. A total of 138 homodesmotic reactions were constructed for 53 compounds of the test set, including 114 HDRs of the first level and 24 HDRs of the second and third levels (Table S2 in Supplementary Materials). It is important to note that the difference between the sums of stoichiometric coefficients for reactants and products is zero for all HDRs. On the basis of these formal reactions, the possibility of applying the homodesmotic approach to the determination of *C_P_* from QC data, as well as using only empirical information for the gas and liquid phases, was studied. Since the ideal balance of a homodesmotic reaction in terms of heat capacity corresponds to Δ*C_P_* = 0, the accuracy of the *C_P_* calculation for the KIAS16 test set was characterized by the mean absolute value of the HDRs heat capacities (MA Δ*C_P_*). It should be noted that MA Δ*C_P_* depends on the reliability of experimental data, which is not always obvious. This is why some *C_P_* values from NIST databases were re-evaluated with the rationale outlined below.

### 3.3. DFT Estimation of Gas-Phase CP at 298 K

The direct calculation of the heat capacity of organic compounds based on the concepts of statistical mechanics and implemented in most quantum chemical software packages can in some cases provide a result with a fairly high accuracy. However, thermochemical QC calculations are inherent in a number of well-known model limitations and problems: the RRHO (rigid rotor–harmonic oscillator) approximation, low-frequency vibration modes, possible equilibrium coexistence of several conformational states of a compound under study, non-valence effects, primarily hydrogen bonding, etc. This leads to systematic deviations of the calculated heat capacity from the experiment in a row of similar structures. These trends may be observed for the KIAS16 test set. Although the whole “calculation-experiment” dependence looks very reliable with a high correlation coefficient (Figure S1), it is easy to see that the systematic correction for different classes of organic compounds is not the same, and even for linear and non-linear alkanes it is different (Figure S2). Complicating the level of theory to improve the quality of QC calculations does not make sense in this case: unlike the energy quantities, variational principle is not directly used in calculating the heat capacity. Indeed, the change in the DFT approximation (B3LYP/6-31G(d) → M06-2X/cc-pVTZ) does not improve the quality of *C_P_* calculations (Figure S1). It allows us to recommend the direct *C_P_* calculation with great caution and the need for mandatory independent control of the theoretical estimates.

On the other hand, the homodesmotic method, based on the principle of maximum similarity between the studied compound and reference structures, should obviously be suitable for the effective compensation of systematic errors in *C_P_* when calculating the change in the heat capacities of the HDR participants. To test this assumption, Δ*C_P_* of all HDRs used in this work were calculated (Table S2), and it was found that in the overwhelming majority of cases, this value is close to zero or, at least, the estimated error of QC calculation (ΔΔ*C_P_*), usually exceeds the value of Δ*C_P_*. The result of statistical treatment of the Δ*C_P_* array for the KIAS16 test set is shown in Table 1.

The Δ*C_P_* distribution has a nearly Gaussian form (Figure S3) with a maximum shifted towards negative values by approximately 0.5 (B3LYP) and 0.8 J/mol·K (M06-2X). The use of HDR2 and HDR3 noticeably improves the heat capacity balance of the homodesmotic reaction. Thus, for 24 HDRs of higher levels, the mean absolute value of Δ*C_P_* calculated in the B3LYP/6-31G(d) approximation is only 0.25 J/mol·K. Moreover, the dropout of three points with Δ*C_P_* ~1 J/mol·K decreases MA Δ*C_P_* to 0.14 J/mol·K, which in terms of the energy scale Δ*C_P_*·*T* corresponds to a vanishingly small 0.04 kJ/mol at 298 K, i.e., HDR2 and HDR3 provide an ideal balance in terms of heat capacity.

Such an impressive result unambiguously indicates the fundamental possibility of applying the homodesmotic method for the reliable estimations of *C_P_* of organic compounds. Unfortunately, this conclusion cannot be attributed to the estimation of the absolute value of *C_P_* based on QC calculations only, since the systematic error that disappears when calculating the Δ*C_P_* of HDR is preserved when calculating the *C_P_* of the studied compound from the heat capacities of the reference structures and the condition *C_P_* = 0. For example, Δ*C_P_* is small (0.54 J/mol·K) for the HDR No.1 (Table S2), which indicates a good balance in terms of heat capacity. However, the calculation of the absolute value *C_P_* for *n*-butane using the *C_P_* of the reference compounds (ethane and propane) is characterized by a large deviation (9.2 J/mol·K) from the experimental value.

### 3.4. Estimation of Gas-Phase C_P_ (298 K) from Experimental Data by the Homodesmotic Method

The performance of heat capacity balance in the KIAS16 homodesmotic reactions, noted above for DFT-calculated *C_P_*, is also observed when using gas-phase experimental isobaric molar heat capacities. The available information array was treated as follows.

First, it turned out during the analysis that information on all participants of the formal processes is available only for 44 HDRs, for which Δ*C_P_* were calculated. It was found that, with two exceptions, the values of Δ*C_P_* are small. The significant deviation of Δ*C_P_* from zero was observed for HDRs, in which methyl ethyl and diethyl ethers are involved as reagents or products. The initially used values of *C_P_* 93.3 (EtOMe) and 119.4 (EtOEt) J/mol·K were calculated using statistical mechanics theory and the necessary set of structural and spectral parameters determined experimentally [48]. Heat capacities for other compounds taken from this compilation [48] along with data of other authors (Table S1) form an internally consistent array, which made it possible to question the reliability of *C_P_* for EtOMe and EtOEt. Note also that the additive scheme of *C_P_* estimation applying to these ethers achieves noticeably different values: 89.7 (EtOMe) and 112.5 (EtOEt) J/mol·K [49]. In this work, we used our estimate, which allows us to restore the self-consistency of the *C_P_* array with both ethers in it: 88 (EtOMe) and 111 (EtOEt) J/mol·K at 298 K. Under this condition, the mean absolute value of Δ*C_P_* is 1.86 J/mol·K for 44 HDRs, which indicates the reliability of the homodesmotic method for choosing a formal process with a balance in terms of heat capacity between products and reagents.

Second, the performance of the Δ*C_P_* balance makes it possible to estimate unknown heat capacity if HDR includes one compound with a missing *C_P_* value. If, at the same time, a multiple estimation of *C_P_* for the studied compound is available using CS HDRs, then it becomes possible to control the quality of calculation results. The analysis of the data array indicates good reproducibility of the results, as illustrated by *C_P_* calculation for pentanone-3 (Table 2). The isobaric molar heat capacities for another 19 organic compounds were calculated in a similar way (Table 3). For a number of structures, including all N-containing compounds, *C_P_* estimates are impossible due to the partial or complete lack of information on the heat capacities of the reference compounds.

Using this combined (experiment plus HDR estimation) description, it was possible to characterize the gas-phase heat capacities of 42 compounds from the KIAS16 test set. Then, the corresponding Δ*C_P_* of HDRs were calculated. The *C_P_* values for five compounds (Table 3) were estimated from a single HDR, so these compounds were excluded from the array when calculating MA Δ*C_P_*. For the remaining 37 compounds described by 112 homodesmotic reactions, the value of MA Δ*C_P_* was found to be 1.44 J/mol·K (Table 4), which corresponds to an error of ~0.4 kJ/mol at 298 K. This result allows us to recommend the homodesmotic method for the reliable estimation of the gas-phase isobaric heat capacities of organic compounds during the thermochemical analysis of chemical processes. We also note that a more stringent similarity control within the HDRs of levels 2 and 3 somewhat improves the balance of the formal process in terms of heat capacity: MA Δ*C_P_* = 1.36 J/mol·K (Table 4).

### 3.5. Estimation of Liquid-Phase C_P_ (298 K) by the Homodesmotic Method

For practical purposes, information on the heat capacities of organic compounds in the liquid state is often needed. Obviously, intermolecular interactions in a condensed medium can disrupt the energy balance of a homodesmotic reaction. Nevertheless, we should expect in similar structures that interactions between molecules will be close in energy; therefore, the homodesmotic method was applied for calculating liquid-phase *C_P_* for the KIAS16 compounds. The available experimental array of heat capacities is incomplete; therefore, a full-fledged data analysis is possible only within a priori estimation of *C_P_* for a number of important reference structures.

First of all, it should refer to compounds that have a boiling point below 298 K. The isobaric molar heat capacities of propane and *n*-butane were estimated from the linear dependence of *C_P_*(C*_n_*H_2*n*+2_) vs. the number of carbon atoms in liquid *n*-alkanes (Figure S4). From the equation *C_P_*(*n*) = (19.8 ± 1.1) + (29.36 ± 0.14)·*n*, *n* = 5–10, *R*^2^ = 0.9999, it was obtained *C_P_*(C_3_H_8_) = 107.9 and *C_P_*(*n*-C_4_H_10_) = 137.2 J/mol·K. The heat capacity of ethane calculated using HDRs 17 and 18 was found to be 75.5 J/mol·K. Further, the *C_P_* value of isobutane was estimated from four HDRs (7, 14.2, 14.3 and 15.2), obtaining an average of 133.5 ± 1.5 J/mol·K. It is important to emphasize that we considered the calculated *C_P_* values of C_2_–C_4_ alkanes as empirical parameters necessary for use in the homodesmotic scheme for calculating the heat capacities of organic compounds. Indeed, the isobaric heat capacities of lower alkanes in the liquefied state at 298 K [50,51] are systematically higher than the above values. With these *C_P_*, the array of hydrocarbons (excepting alkynes) characterized by MA Δ*C_P_* equals to 3.18 J/mol·K for 26 HDRs.

In the case of carbonyl compounds, an important reference structure is acetaldehyde, *T*_boil_ = 294 K. The *C_P_* value of 89.05 J/mol·K taken from [47] is apparently inaccurate, since it leads to significant deviations of Δ*C_P_* from zero for HDRs with the participation of aldehyde. We used Δ*C_P_*(MeCHO) = 102 J/mol·K, which restores the reproducibility of results for the CS HDRs 41 and 42. In addition, the chosen value agrees reasonably with the *C_P_* value of 96.2 J/mol·K for 273 K [52].

In the group of alcohols and ethers, the key reference structures are dimethyl ether (*T*_boil_ = 248 K) and methyl ethyl ether (*T*_boil_ = 283 K). The liquid-phase heat capacities of these ethers at 298 K were calculated using HDRs 19 and 28.3:

(19) EtOMe + MeOH → MeOMe + EtOH,

(28.3) MeOCH_2_CH_2_OMe + C_2_H_6_ + MeOH → MeOMe + EtOH + EtOMe

Using the known heat capacities of reference compounds, one can find the difference of *C_P_* for methyl ethyl and dimethyl ethers from the first equation, 31.1 J/mol·K, and their sum from the second one, 236.6 J/mol·K, where *C_P_*(MeOMe) = 102.75 J/mol·K and *C_P_*(EtOMe) = 133.85 J/mol·K. In addition, the isobaric molar heat capacity of diethyl ether [47] seems to be overestimated. This conclusion follows from a comparison with *C_P_* for isomeric methyl propyl ether (Table S1) and the scale of change in heat capacity in the homologous series of ethers with an increase in the length of the main chain by a CH_2_ group. The corrected value *C_P_*(EtOEt) = 165.35 J/mol·K was found as the sum of *C_P_*(EtOMe) and the contribution of CH_2_ group to the heat capacity of ethers, calculated as the difference between the heat capacities of PrOEt and PrOMe, as well as EtOMe and MeOMe. Formally, this corresponds to the calculation of *C_P_* using HDRs of level 3 and 2, respectively:

EtOEt + PrOMe → EtOMe + PrOEt, EtOEt + MeOMe → 2 EtOMe

Further, the analysis of the HDR array was carried out similarly to that described above for gas-phase heat capacities. In addition to those mentioned above, thirteen more *C_P_* were estimated by the homodesmotic method (Table 5), and the reaction heat capacities of 102 HDRs were calculated for 32 compounds of the test set. The value MA Δ*C_P_* = 2.83 J/mol·K (Table 4) is approximately two times higher than that for gas-phase HDRs. Nevertheless, the energy imbalance of heat capacities Δ*C_P_*·*T* remains less than 1 kJ/mol, which indicates the fundamental applicability of the homodesmotic method to liquid-phase systems. It is less obvious for alcohols with branched carbon chain due to the rather high values of Δ*C_P_* (Table S4).

### 3.6. Gas-Phase Heat Capacities of n-Alkanes at 200–1500 K

The heat capacities of linear alkanes C_2_–C_10_ were taken from the database [46], while the recommended *C_P_* values in the range of 200–1500 K were used. The *C_P_* arrays for all *n*-alkanes were described within the framework of the Shomate equation:

*C_P_* = *a*_0_ + *a*_1_*τ* + *a*_2_*τ*^2^ + *a*_3_*τ*^3^ + *a*_−2_*τ*^−2^, (1)

where *τ* = T/298.15 is dimensionless temperature; *a*_i_, the coefficients of the Shomate equation (Table S3), have the same dimension of heat capacity. Equation (1) describes the *C_P_* arrays with high accuracy (MA deviation of 0.43 J/mol·K). Table S3 also lists the standard enthalpy of formation Δ*_f_H*° [45], the absolute enthalpy *H*°, and the isobaric molar heat capacity *C_P_* at 298.15 K of *n*-alkanes calculated in the B3LYP/GTBas3 approximation using the Gaussian-09 program [36]. This level of theory is used in the G4 composite method to optimize the structure and calculate the vibrational frequencies of molecules.

Further, complete sets of first level HDRs for C_4_–C_10_ *n*-alkanes were constructed in accordance with the procedure described in [8,30,31]. The reaction coefficients of Shomate equation for each HDR were calculated according to its stoichiometry (Table S4). It is important to note that the homodesmotic condition Δ*a*_i_ = 0 must also be met for the entire set of Shomate equation coefficients. Although the tendency towards zeroing of the coefficients is quite pronounced, a growing deviation of Δ*a*_i_ from zero is observed with an increase in the length of the alkane carbon chain. Obviously, this is partly due to the error in determining *C_P_*. It may suggest also that some deterioration in the fulfillment of the thermoneutrality condition takes place for HDR1 of higher alkanes. This effect is almost indistinguishable when calculating Δ*_r_H*° of HDRs using experimental enthalpies of formation of *n*-alkanes, since it is small and comparable to the error of Δ*_f_H*° determination. The heat effect of HDRs calculated in the B3LYP/GTBas3 approximation weakly but systematically varies: 0.17–0.51 for hexane, 0.26–1.27 for octane and 0.15–1.89 for decane, all values are shown in kJ/mol. We believe that the reason for the observed effect is the difference in the far environment of the atoms in the HDRs used. Indeed, the only co-reagent in HDR1 is ethane (Table S4), having two thermochemical groups of the form [C-(CH_3_)(H)_3_]. There is no such group in the right side of HDRs. The terminal carbon atoms in the products have an environment of [C-(CH_2_C)(H)_3_]. In addition, the propane molecule has an unique environment of the secondary carbon atom [C-(CH_3_)_2_(H)_2_], while in *n*-alkanes, starting from butane, this atom has a slightly different second environment [C-(CH_3_)(CH_2_C)(H)_2_]. We assumed that the effect of small molecules noted in [23] was responsible for the observed drift of Δ*_r_H*°. To test this assumption, the HDR1 thermal effect was considered as a function of the number of small molecules involved in this formal process:

Δ*_r_H*° = Δ*_r_H*°_0_ − *b*_2_*n*_2_ + *b*_3_*n*_3_ + *b*_4_*n*_4_, (2)

where Δ*_r_H*°_0_ is the HDR enthalpy, free from the effect of small molecules; *n*_2_, *n*_3_ and *n*_4_ are the number of ethane, propane and *n*-butane molecules participating in the HDR, respectively; *b*_2_, *b*_3_ and *b*_4_ are the enthalpy corrections characterizing the effect of small molecules.

The entire array of HDRs for *n*-alkanes C_4_–C_10_ is excellently described within the framework of Equation (2). It was found (correlation coefficient *R* = 0.98):

Δ*_r_H*°_0_ = −0.02 ± 0.03, *b*_2_ = 0.70 ± 0.03,

*b*_3_ = 0.39 ± 0.02, *b*_4_ = 0.04 ± 0.02,

where the standard error of approximation is 0.10; all values are shown in kJ/mol. It should be noted that dependence (2) is also preserved for thermal effects calculated by the G4 composite method.

The results of the regression analysis allow us to draw the following conclusions:

- All HDRs are thermoneutral, taking into account the effect of small molecules;
- An increase in the size of a small molecule, as expected, diminishes the enthalpy correction to almost zero for *n*-butane;
- “Ideal” HDRs for *n*-alkanes should be compiled with reference compounds not less than *n*-butane.

Note that the last conclusion corresponds to the use of homodesmotic reactions of the third level.

**Figure 2:**
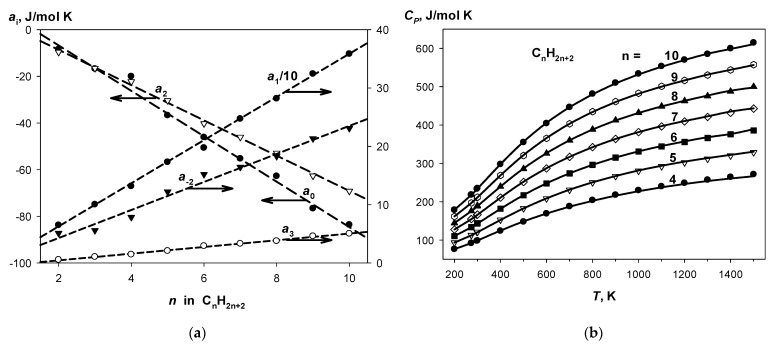
(**a**) Coefficients of the Shomate equation for *n*-alkanes. (**b**) Comparison of isobaric molar heat capacities calculated using the CS HDR3 with the recommended data.

One may see that to fulfill the condition Δ*a*_i_ = 0, it is necessary and sufficient to have linear dependences of the coefficients of Shomate equation in the series of *n*-alkanes. Indeed, these dependences are really observed with high correlation coefficients; see Figure 2a. It allows us to obtain corrected optimal values of *a*_i_ fitted the homodesmotic conditions (see Table 6 for C_4_–C_10_ alkanes). The complete sets of HDR3 for *n*-alkanes from hexane to decane are shown in Table S6. Thus, an internally consistent set of C_4_–C_10_ alkane isobaric hear capacities was obtained, which is in good agreement with the recommended dataset [46], as shown in Figure 2b. The homodesmotic method provides the mean absolute deviation from the recommended *C_P_* values of 0.93 J/mol·K for the entire temperature range (200–1500 K) with maximum error of 3.5 J/mol·K, which reaches 5 kJ/mol in energy units (*C_P_·T*) at high temperatures.

If the level 3 of HDRs cannot be applied to a test compound, then HDR1 and HDR2 are viable options. The comparison of the results obtained with HDR3 vs. HDR1 or 2 (Table 6, Tables S5 and S6; Figure S5) indicates that the HDR3 method works better at high temperatures, whereas the homodesmotic reactions of all levels provide similar results up to ~700–800 K.

## 4. Conclusions

The homodesmotic principle of formal process design based on the maximum similarity of reagents and products ensures the fulfillment of a number of balance conditions, which, in turn, cause thermoneutrality of the formal process or, at least, a weak temperature dependence of reaction enthalpy Δ*_r_H*°. A thermodynamic consequence of the independence of HDR enthalpy from temperature is zero or near-zero heat capacity of the reaction. The group balance of HDR, i.e., the similarity of the nearest and more distant environments of atoms (in the case of HDR2 and higher levels), as well as the isostoichiometric nature of HDR (Δν = 0) cause the same character of the internal motion in molecules-reagents and products of HDR, which reflects in the near-equal sums of partition functions and, therefore, leads to the reaction heat capacity close to zero. The results of QC calculations quantitatively confirm this conclusion (Table 1). Since it is possible for a correctly chosen HDR to assign with a high degree of confidence the reaction heat capacity Δ*C_P_* as zero, this opens up the possibility of theoretical prediction of the absolute value of isobaric molar heat capacity of a compound from known *C_P_* values for all other reaction participants. Here, it is necessary to answer the question: how to choose the right type of formal process? The results of this study show that, from a practical point of view, even the first level of HDRs provides an acceptable level of reliability of *C_P_* estimation in most cases. Moreover, the use of complete sets of HDRs for the compound under study makes it possible to control the reproducibility of the *C_P_* calculation and, if necessary, to justify the rejection of unreliable heat capacities of the reference compounds. On the other hand, the HDR1 results can be verified using higher levels of HDRs, if the size of studied compound and the availability of *C_P_* for reference structures allow this verification. Since there is a tempting prospect of using quantum chemical calculations of *C_P_* in the absence of heat capacity for a reference compound, it is noteworthy that the QC-determined heat capacities should be applied with caution due to possible significant systematic error from the model limitations used in the calculation (RRHO approximations, single-conformer description, non-valence effects, etc.).

The most reliable estimates of *C_P_* are possible for gas-phase heat capacities. The data analysis of *C_P_* at 298 K for compounds of the KIAS16 test set and for *n*-alkanes in a wide temperature range showed that the mean error of *C_P_* estimation does not exceed 1.5 J/mol·K. The isobaric molar heat capacities of 20 organic compounds were estimated using the homodesmotic method (Table 3). The coefficients of the Shomate equation were obtained for *n*-alkanes, which ideally satisfy the homodesmotic condition and perfectly describe the heat capacities in the range of 200–1500 K. Note that the accuracy of describing the array of heat capacities slightly but systematically increases with the use of HDR of a higher level: MAD = 1.29 (HDR1), 1.21 (HDR2) and 0.93 (HDR3) J/mol·K. Homodesmotic reactions of the first and second levels are not inferior to HDR3 up to temperatures ~700–800 K.

The expected result was obtained from the analysis of the liquid-phase heat capacities for the KIAS16 compounds: the HDR balance in terms of heat capacity was well performed, but the specific intermolecular interactions in condensed medium worsened the *C_P_* balance by about two times. In addition, “bad” compounds were identified, for which HDR Δ*C_P_* is unacceptably high (branched alcohols). It should also be noted that the HDR design methodology uses a number of reference compounds that have a gas-liquid phase transition temperature below 298 K. For such compounds, empirical values of liquid-phase heat capacities were selected, the physical meaning of which is poorly defined. However, this shortcoming can be overcome, for example, by using higher levels of HDRs. For a number of compounds, the liquid phase *C_P_* was evaluated (Table 5).

The reliability of homodesmotic estimates of *C_P_* can also be illustrated as follows. Acree and Chickos have recently showed the existence of fair correlation between heat capacities of organic compounds in different phases [53]. The KIAS16 test set, together with reference compounds, includes 80 structures, for 53 of them the isobaric molar heat capacities are known or determined in this work both in the gas and liquid phases. Indeed, these *C_P_* values are linearly related (Figure S6):

*C_P_*(gas) = (0.72 ± 0.04)·*C_P_*(liquid) − (11.2 ± 6.2) J/mol·K (3)

with a correlation coefficient *R* = 0.94. Within the error limits, the regression parameters coincide with the coefficients of Equation (3) shown in [53], −0.74 and 10.58, respectively.

In general, the results of this study allow us to draw the confident conclusion that the homodesmotic method is quite applicable for the a priori estimation of the isobaric molar heat capacities of organic compounds of various classes; and the author of the work expresses the hope that the proposed approach will take its place in the arsenal of modern theoretical thermochemistry for solving scientific and practical problems of organic chemistry.

## Figures and Tables

**Table 1:** DFT-calculated mean absolute heat capacity (MA Δ*C_P_*, J/mol·K) for the HDRs of the KIAS16 test set, 298 K.

Group of Compounds ^1^	Number of HDRs	B3LYP/6-31G(d)	M06-2X/cc-pVTZ
Hydrocarbons (16)	44	0.53	1.64
Alcohols and ethers (20)	60	1.04	2.00
Carbonyl compounds (7)	14	0.53	0.91
N-containing (10)	20	0.44	1.27
KIAS16 test set (53)	138	0.74	1.67
including HDR2 and 3	24	0.25	0.78

^1^ In parentheses, the total number of compounds in the set.

**Table 2:** CS HDR estimation of the gas-phase isobaric molar heat capacity of pentanone-3, J/mol·K, 298 K ^1^.

Level of HDR	Homodesmotic Reaction	*C_P_*
1	EtC(O)Et + MeCHO → EtC(O)Me + EtCHO	127.1
1	EtC(O)Et + 2 MeCHO → MeC(O)Me + 2 EtCHO	125.8
2	EtC(O)Et + EtCHO → EtC(O)Me + PrCHO	124.3
2	EtC(O)Et + 2 MeC(O)Me → 2 EtC(O)Me	128.3
Average:	*C_P_* (298 K) of pentanone-3 is	126.4 ± 1.7

^1^ Assuming Δ*C_P_* = 0. Heat capacities of reference compounds are shown in Table S1.

**Table 3:** Gas-phase isobaric molar heat capacities *C_P_*, J/mol·K, 298 K estimated by the homodesmotic method ^1^.

No.	Compound	*C_P_*	Comment
1	EtCHO	85.5	Equation 38
2	MeOCH_2_CH_2_OH	100.1 ± 0.7	Equations 20.1–20.4
3	Me_2_CHCH_2_OH	108.6	Equation 22
4	PrOMe	108.1 ± 0.9	Equations 24.1–24.4
5	Me_2_CHOMe	112.1	Equation 26
6	HO(CH_2_)_4_OH	120.6 ± 2.0	Equations 27.1–27.5
7	MeOCH_2_CH_2_OMe	121.9 ± 1.1	Equations 28.1–28.5
8	MeC(O)Pr	123.7 ± 1.9	Equations 41.1–41.5
9	EtC(O)Et	126.4 ± 1.7	Equations 42.1–42.4
10	Me(CH_2_)_4_OH	130.3 ± 2.1	Equations 29.1–29.6
11	Me_2_CHCH_2_CH_2_OH	132.3 ± 1.7	Equations 30.1–30.3
12	EtMeCHCH_2_OH	132.6 ± 1.6	Equations 31.1–31.3
13	PrMeCHOH	135.0 ± 1.0	Equations 32.1–32.3
14	Me_2_CHCH(Me)OH	133.2	Equation 33
15	BuOMe	130.4 ± 2.2	Equations 34.1–34.7
16	PrOEt	129.7 ± 1.3	Equations 35.1–35.6
17	Me_3_COMe	136.4	Equation 36
18	EtC≡CEt	120.6 ± 0.4	Equations 10.1–10.4
19	BuCH=CH_2_	130.5 ± 2.0	Equations 11.1–11.5
20	Et_2_C=CH_2_	131.6 ± 0.9	Equations 12.1–12.4

^1^ See Table S2 for HDRs.

**Table 4:** Mean absolute value of HDR heat capacities (MA Δ*C_P_*, J/mol·K) determined on the base of experimental data, 298 K ^1,2,3^.

Group of Compounds	Gas		Liquid	
	Number of HDRs	MA Δ*C_P_*	Number of HDRs	MA Δ*C_P_*
Hydrocarbons	44 (16)	1.66	26 (9)	3.18
Alcohols and ethers	56 (16)	1.23	47 (11)	2.17
Carbonyl compounds	12 (5)	1.59	13 (6)	3.80
N-containing	–	–	16 (6)	3.37
KIAS16 test set	112 (37)	1.44	102 (32)	2.83
including HDR2 and HDR3	20	1.36	18	2.68

^1^ Excluding *C_P_* estimated with a single HDR, since Δ*C_P_* is equal to zero on definition in this case; ^2^ If HDR has two or more missing *C_P_*, it was excluded from the analysis; ^3^ In parentheses, the total number of compounds in the set.

**Table 5:** Liquid-phase isobaric molar heat capacities *C_P_*, J/mol·K, 298 K estimated by the homodesmotic method ^1^.

No.	Compound	*C_P_*	Comment
1	MeCHO	102	Equations 40; 41.1,3; 42.1,2
2	MeC(O)NH_2_	94.0	Equation 45
3	MeOMe	102.8	Equations 19; 28.3
4	C_2_H_6_	75.5	Equations 17; 18
5	EtNH_2_	129.4 ± 6.2	Equations 44; 47.2,3,5
6	EtC(O)NH_2_	126.7	Equation 51
7	EtOMe	133.9	Equations 19; 28.3
8	C_3_H_8_	107.9	Liquid alkanes regression
9	Et_2_CHCHO	156.1	Equation 43
10	Me_2_C=CH_2_	130.2	Equation 5
11	PrC(O)NH_2_	158.1 ± 1.7	Equations 50.1–50.3
12	EtOEt	165.4	See text
13	*n*-C_4_H_10_	137.2	Liquid alkanes regression
14	Me_3_CH	133.5 ± 1.5	Equations 7; 14.1,2; 15.2
15	EtMeCHNH_2_	193.7	Equation 49
16	BuC≡N	166.1 ± 2.9	Equations 52.1–52.5
17	EtMeCHC≡N	185.5	Equation 53
18	EtMeCHCH_2_OH	203.9 ± 6.0	Equations 31.1–31.3
19	PrMeCHOH	225.0 ± 4.0	Equations 32.1–32.3
20	Me_4_C	159.0	Equation 16
21	Et_2_C=CH_2_	183.9 ± 0.6	Equations 12.1–12.4

^1^ See Table S2 for HDRs.

**Table 6:** Isobaric molar heat capacities of *n*-alkanes (J/mol·K).

Compound	*a*_0_	*a*_1_	*a*_2_	*a*_3_	*a*_−2_	*C_P_* (298K) ^1^	*C_P_* (calc) ^2^
Ethane	−8.48	64.96	−9.63	0.56	5.10	52.49	52.5
Propane	−16.75	100.42	−16.54	1.07	5.59	73.60	73.8
Butane ^3^	−26.19	136.84	−23.75	1.61	9.10	98.49	97.6
Pentane ^3^	−35.92	173.84	−31.30	2.19	11.50	120.0 ± 0.1	120.3
Hexane ^3^	−45.66	210.85	−38.86	2.76	13.90	142.6 ± 0.2	143.0
Heptane ^3^	−55.40	247.86	−46.42	3.33	16.31	165.2 ± 0.3	165.7
Octane ^3^	−65.13	284.87	−53.98	3.91	18.71	187.8 ± 0.4	188.4
Nonane ^3^	−74.87	321.88	−61.53	4.48	21.12	210.4 ± 0.5	211.1
Decane ^3^	−84.61	358.89	−69.09	5.06	23.52	233.1 ± 0.6	233.8

^1^ See Table S2 for HDRs. ^2^ As sum of ai; ^3^ The Shomate equation coefficients were corrected in accord with linear dependence for C_4_-C_10_ (Figure 2).

## Data Availability

Data are contained within the article or supplementary material.

## Supplementary Materials

The following supporting information can be downloaded at: https://www.mdpi.com/article/10.3390/molecules27227814/s1, Figures S1 and S2: Direct *C_P_* calculation; Figure S3: Δ*C_P_* distribution; Figure S4: Isobaric molar heat capacities for liquid *n*-alkanes; Figure S5: Isobaric heat capacities for *n*-alkanes from HDR 1 and 2; Figure S6: Acree–Chickos correlation of isobaric heat capacities; Table S1: Isobaric molar heat capacities for the test set of compounds; Table S2: Complete sets of HDRs; Table S3: Coefficients of Shomate equation and calculated absolute enthalpy and heat capacity for linear alkanes; Table S4: Complete set of HDRs 1 for *n*-alkanes; Table S5: Complete set of HDRs 2 for *n*-alkanes; Table S6: Complete set of HDRs 3 for *n*-alkanes.

## Sample Availability

Samples of the compounds are not available from the authors.

## References

[1] Benson S.W., Buss J.H. (1958). Additivity rules for the estimation of molecular properties. Thermodynamic properties. J. Chem. Phys..

[2] Benson S.W. (1976). Thermochemical Kinetics: Methods for the Estimation of Thermochemical Data and Rate Parameters.

[3] Hehre W.J., Ditchfield R., Radom L., Pople J.A. (1970). Molecular orbital theory of the electronic structure of organic compounds. V. Molecular theory of bond separation. J. Am. Chem. Soc..

[4] George P., Trachtman M., Bock C., Brett A.M. (1975). An alternative approach to the problem of assessing stabilization energies in cyclic conjugated hydrocarbons. Theor. Chim. Acta.

[5] George P., Trachtman M., Brett A.M., Bock C. (1977). Comparison of various isodesmic and homodesmotic reaction heats with values derived from published ab initio molecular orbital calculations. J. Chem. Soc. Perkin Trans. 2.

[6] Wheeler S.E., Houk K.N., Schleyer P.v.R., Allen W.D. (2009). A hierarchy of homodesmotic reactions for thermochemistry. J. Am. Chem. Soc..

[7] Wheeler S.E. (2012). Homodesmotic reactions for thermochemistry. WIREs Comput. Mol. Sci..

[8] Khursan S.L., Ismagilova A.S., Akhmerov A.A., Spivak S.I. (2016). Constructing homodesmic reactions for calculating the enthalpies of formation of organic compounds. Russ. J. Phys. Chem. A.

[9] Novak I. (2016). Computational thermochemistry of C-nitroso compounds. Struct. Chem..

[10] Song G., Bozzelli J.W. (2017). Structural and thermochemical studies on CH_3_SCH_2_CHO, CH_3_CH_2_SCHO, CH_3_SC(=O)CH_3_, and radicals corresponding to loss of H atom. J. Phys. Org. Chem..

[11] Song G., Bozzelli J.W. (2018). Structures and thermochemistry of methyl ethyl sulfide and its hydroperoxides: HOOCH_2_SCH_2_CH_3_, CH_3_SCH(OOH)CH_3_, CH_3_SCH_2_CH_2_OOH, and radicals. J. Phys. Org. Chem..

[12] Song G., Bozzelli J.W. (2018). Structural and thermochemical properties of methyl ethyl sulfide alcohols: HOCH_2_SCH_2_CH_3_, CH_3_SCH(OH)CH_3_, CH_3_SCH_2_CH_2_OH, and radicals corresponding to loss of H atom. J. Phys. Org. Chem..

[13] Zhao Y., Cheng X., Nie K., Han Y., Li J. (2021). Structures, relative stability, bond dissociation energies, and stabilization energies of alkynes and imines from a homodesmotic reaction. Comput. Theor. Chem..

[14] Dorofeeva O.V., Ryzhova O.N. (2021). Accurate estimation of enthalpies of formation for C-, H-, O-, and N-containing compounds using DLPNO-CCSD(T1)/CBS method. Struct. Chem..

[15] Poskrebyshev G.A. (2021). The standard thermochemical properties of the p-benzylphenol and dimethyl phthalate, and their temperature dependencies. Comput. Theor. Chem..

[16] Poskrebyshev G.A. (2022). The values of Δ_f_H^o^_298.15_ and S^o^_298.15_ of the radicals formed by the abstraction of H atom from the p-benzylphenol and dimethyl phthalate. Int. J. Chem. Kinet..

[17] Alonso M., Herradón B. (2010). A universal scale of aromaticity for π-organic compounds. J. Comput. Chem..

[18] An K., Zhu J. (2018). Direct energetic evaluation of aromaticity by cleaving the rings of cyclic compounds. J. Organomet. Chem..

[19] Szatylowicz H., Jezuita A., Krygowski T.M. (2019). On the relations between aromaticity and substituent effect. Struct. Chem..

[20] Magers D.B., Magers A.K., Magers D.H. (2019). The s-homodesmotic method for the computation of conventional strain energies of bicyclic systems and individual rings within these systems. Int. J. Quantum. Chem..

[21] Watanabe K., Segawa Y., Itami K. (2020). A theoretical study on the strain energy of helicene-containing carbon nanobelts. Chem. Commun..

[22] Akhmetshina E.S., Khursan S.L. (2020). Application of group separation reaction formalism for analysis of non-valence effects of organic compounds: Three-carbon rings. Russ. Chem. Bull..

[23] Akhmetshina E.S., Khursan S.L. (2020). Complete set of homodesmotic reactions for the analysis of non-valence effects in the three-to-six-membered cyclic organic compounds. Thermochim. Acta.

[24] Khursan S.L., Akhmetshina E.S. (2021). Interplay of the ring and steric strains in the highly substituted cyclopropanes. J. Phys. Chem. A.

[25] Fokin A.A., Reshetylova O.K., Bakhonsky V.V., Pashenko A.E., Kivernik A., Zhuk T.S., Becker J., Dahl J.E.P., Carlson R.M.K., Schreiner P.R. (2022). Synthetic doping of diamondoids through skeletal editing. Org. Lett..

[26] Planells A.R., Ferao A.E. (2022). Accurate ring strain energies of unsaturated three-membered heterocycles with one group 13–16 element. Inorg. Chem..

[27] Planells A.R., Ferao A.E. (2022). Ring strain energies of three-membered homoatomic inorganic rings El_3_ and diheterotetreliranes El_2_Tt (Tt = C, Si, Ge): Accurate versus additive approaches. Inorg. Chem..

[28] Fishtik I., Datta R. (2003). Group additivity vs ab initio. J. Phys. Chem. A.

[29] Verevkin S.P., Emel’yanenko V.N., Diky V., Muzny C.D., Chirico R.D., Frenkel M. (2013). New group-contribution approach to thermochemical properties of organic compounds: Hydrocarbons and oxygen-containing compounds. J. Phys. Chem. Ref. Data.

[30] Khursan S.L., Ismagilova A.S., Spivak S.I. (2017). A graph theory method for determining the basis of homodesmic reactions for acyclic chemical compounds. Dokl. Phys. Chem..

[31] Khursan S.L., Ismagilova A.S., Ziganshina F.T., Akhmet’yanova A.I. (2021). Constructing a complete set of homodesmic reactions using the depth-first search procedure. Russ. J. Phys. Chem. A.

[32] Nguyen H.T., Mai T.V.-T., Huynh L.K. (2019). mHDFS-HoF: A generalized multilevel homodesmotic fragment-separation reaction based program for heat-of-formation calculation for acyclic hydrocarbons. J. Comput. Chem..

[33] Minenkova I., Otlyotov A.A., Cavallo L., Minenkov Y. (2022). Gas-phase thermochemistry of polycyclic aromatic hydrocarbons: An approach integrating the quantum chemistry composite scheme and reaction generator. Phys. Chem. Chem. Phys..

[34] McQuarrie D.A., Simon J.D. (1999). Molecular Thermodynamics.

[35] Acree W.E., Chickos J.S. (2021). JPCRD: 50 years of providing the scientific community with critically evaluated thermodynamic data, predictive methods, and large thermodynamic data compilations. J. Phys. Chem. Ref. Data.

[36] Frisch M.J., Trucks G.W., Schlegel H.B., Scuseria G.E., Robb M.A., Cheeseman J.R., Scalmani G., Barone V., Mennucci B., Petersson G.A. (2016). Gaussian 09.

[37] Becke A.D. (1993). Density-functional thermochemistry. III. The role of exact exchange. J. Chem. Phys..

[38] Lee C., Yang W., Parr R.G. (1988). Development of the Colle-Salvetti correlation-energy formula into a functional of the electron density. Phys. Rev. B.

[39] Ditchfield R., Hehre W.J., Pople J.A. (1971). Self-consistent molecular-orbital methods. IX. An extended gaussian-type basis for molecular-orbital studies of organic molecules. J. Chem. Phys..

[40] Zhao Y., Truhlar D.G. (2008). The M06 suite of density functionals for main group thermochemistry, thermochemical kinetics, noncovalent interactions, excited states, and transition elements: Two new functionals and systematic testing of four M06-class functionals and 12 other functionals. Theor. Chem. Acc..

[41] Dunning T.H. (1989). Gaussian basis sets for use in correlated molecular calculations. I. The atoms boron through neon and hydrogen. J. Chem. Phys..

[42] Curtiss L.A., Redfern P.C., Raghavachari K. (2007). Gaussian-4 theory. J. Chem. Phys..

[43] Růžička V., Domalski E.S. (1993). Estimation of the heat capacities of organic liquids as a function of temperature using group additivity. I. Hydrocarbon compounds. J. Phys. Chem. Ref. Data.

[44] Zábranský M., Růžička V. (2004). Estimation of the heat capacities of organic liquids as a function of temperature using group additivity: An amendment. J. Phys. Chem. Ref. Data.

[45] Afeefy H.Y., Liebman J.F., Stein S.E., Linstrom P.J., Mallard W.G. Neutral thermochemical data. NIST Chemistry Webbook, NIST Standard Reference Database Number 69.

[46] Linstrom P.J., Mallard W.G. Entropy and heat capacity of organic compounds by Glushko thermocenter, Russian academy of sciences, Moscow. NIST Chemistry Webbook, NIST Standard Reference Database Number 69.

[47] Domalski E.S., Hearing E.D., Linstrom P.J., Mallard W.G. Condensed phase heat capacity data. NIST Chemistry Webbook, NIST Standard Reference Database Number 69.

[48] Chao J., Hall K.R., Marsh K.N., Wilhoit R.C. (1986). Thermodynamic properties of key organic oxygen compounds in the carbon range C1 to C4. Part 2. Ideal gas properties. J. Phys. Chem. Ref. Data.

[49] Stull D.R., Westrum E.F., Sinke G.C. (1969). The Chemical Thermodynamics of Organic Compounds.

[50] Engineering Toolbox Propane—Specific Heat vs. Temperature and Pressure. https://www.engineeringtoolbox.com/specific-heat-capacity-propane-Cp-Cv-isobaric-isochoric-d_2060.html.

[51] Engineering Toolbox Butane—Specific Heat vs. Temperature and Pressure. https://www.engineeringtoolbox.com/butane-C4H10-specific-heat-capacity-Cp-Cv-isobaric-isochoric-d_2087.html.

[52] Conner A.Z., Elving P.J., Steingiser S. (1947). Specific heats of acetaldehyde and acetaldehyde dibutyl acetal. J. Am. Chem. Soc..

[53] Acree W., Chickos J.S. (2016). Phase transition enthalpy measurements of organic and organometallic compounds. Sublimation, vaporization and fusion enthalpies from 1880 to 2015. Part 1. C1—C10. J. Phys. Chem. Ref. Data.

