# Supplementary material for: Heat Capacity Estimation Using a Complete Set of Homodesmotic Reactions for Organic Compounds
Source: Molecules. 2022 Nov 13;27(22):7814. doi: 10.3390/molecules27227814 (PMC9692375; doi:10.3390/molecules27227814)
Supplement: Supplementary file 1 [file molecules-27-07814-s001.zip › molecules-2023666-supplementary.pdf]

Heat Capacity Estimation Using Complete Set of Homodesmotic Reactions for Organic Compounds

Khursan S.L. [1,*]

[1] Ufa Institute of Chemistry of Ufa Federal Research Center of Russian Academy of Sciences, 71 Prospect Oktyabrya, 450054 Ufa, Russian Federation.
Tel/Fax: +7 (347) 2356066.
* KhursanSL@anrb.ru

SUPPORTING INFORMATION

Content

	Page
**Table S1. Isobaric molar heat capacities for the test set of compounds**	S2
**Table S2. Complete sets of HDRs**	S4
**Figure S1. Direct C_P calculation (exp vs. calc)**	S12
**Figure S2. Direct C_P calculation (various classes)**	S12
**Figure S3. ΔC_P distribution (B3LYP calculations)**	S13
**Figure S4. Isobaric molar heat capacities for liquid *n*-alkanes**	S13
**Table S3. Coefficients of Shomate equation and calculated absolute enthalpy and heat capacity for linear alkanes**	S14
**Table S4. Complete set of HDRs 1 for *n*-alkanes**	S15
**Table S5. Complete set of HDRs 2 for *n*-alkanes**	S19
**Table S6. Complete set of HDRs 3 for *n*-alkanes**	S21
**Figure S5. Isobaric heat capacities for *n*-alkanes from HDR 1 and 2**	S23
**Figure S6. Acree-Chickos correlation of isobaric heat capacities**	S24

Table S1. Isobaric molar heat capacities for the test set of compounds

No.	Compound	Cp (NIST)	Error	Comment	Cp(est)	Error	Comment	Cp(B3LYP)	Cp(M06-2X)	Cp(NIST)liquid	Error	Comment	Cp(est)liquid	Error	Comment
1	CH(O)-OH	45.68	0.07	Chao_86 rcmd				44.19	44.10	98.6	0.67	av. of 2 points			
2	CH3-OH	44.06	0.03	Chao_86 rcmd				44.61	45.31	81.2	1.17	av. of 14 points			
3	CH(O)-CH3	55.32	0.08	Chao_86 rcmd				54.96	54.94	89.05		1 point	102		Correction of 13 J/mol K from eqs. 40; 41.1; 41.3; 42.1; 42.2
4	CH3-C(O)-OH	63.44	0.11	Chao_86 rcmd				67.80	67.31	123.1		1 point			
5	CH(O)-O-CH3	64.38	0.09	Chao_86 rcmd				64.97	64.08	120.1	0.62	av. of 2 points			
6	CH3-C(O)-NH2							77.15	77.29				94.0		eq. 45
7	CH3-CH2-OH	65.21	0.14	Chao_86 rcmd				64.25	64.62	112.3	0.54	av. of 18 points			
8	CH3-O-CH3	65.57	0.08	Chao_86 rcmd				62.03	61.60				102.8		Combination of eqs. 19 and 28.3
9	HO-CH2-CH2-OH	77.99		Yeh_94_rcmd			82.7(0.13) Chao_86	76.12	76.71	149.2	1.05	av. of 6 points			
10	CH3-CH3	52.49		Gurvich_89 rcmd				50.11	50.76				75.5	0.1	av. over eq. 17 and 18
11	CH3-CH2-NH2							67.38	67.75				129.4	6.2	av. over 4 eqs. 44; 47.2; 47.3; 47.5
12	CH3-CCH	60.73		TRC_97 rcmd				60.47	58.70						
13	CH3-CH2-CN							70.64	70.39	105.3		1 point. Another value of 119.5 is doubtful			
14	CH3-C(O)-CH3	75.02	0.11	Chao_86 rcmd				78.49	77.99	125.1	1.1	av. of 10 points			
15	CH(O)-CH2-CH3	80.73	0.1	Chao_86 rcmd				74.34	74.36	134.7		1 point. Another value of 159.1 is doubtful			
16	CH3-C(O)-O-CH3	86.03	0.12	Chao_86 rcmd				89.35	88.90	140.8	0.44	av. of 3 points			
17	CH(O)-O-CH2-CH3				85.5		eq. 38	85.42	84.49	144.3		1 point			
18	CH3-CHCH2	64.32		TRC_97 rcmd				62.32	62.66	102		1 point			
19	CH3-CH2-C(O)-NH2							96.74	97.00				126.7		eq. 51
20	CH3-CH2-CH2-OH	85.56	0.14	Chao_86 rcmd				84.43	84.88	144.6	1.1	av. of 13 points			
21	CH3-CH2-O-CH3	93.3	0.11	Chao_86 rcmd, bad value?	88.0		My estimation. Stull add.scheme gives 89.7	82.53	82.61				133.9		Combination of eqs. 19 and 28.3
22	CH3-CH(CH3)-OH	89.32	0.15	Chao_86 rcmd				87.02	86.64	161.9	1.62	av. of 4 points			
23	CH3-O-CH2-CH2-OH				100.1	0.7	av. over eqs. 20.1 - 20.4	95.45	94.64	175.6	0.88	av. of 3 points			
24	CH3-CH2-CH3	73.6		Chao_86 rcmd				69.71	69.29				107.9		Regression for linear alkanes: 19.80 +- 1.1 + 29.36 +- 0.14 x n
25	CH3-CH2-CH2-NH2							87.37	87.82	162.8	2.65	av. of 4 points			
26	CH3-CH(CH3)-NH2							90.01	90.37	164.4	0.8	av. of 3 points			
27	CH3-CH2-CCH	81.82		TRC_97 rcmd				79.65	77.47						
28	CH3-CC-CH3	78.02		TRC_97 rcmd				80.78	80.28						
29	CH3-CH2-CH2-CN							90.82	90.61	134.2		1 point			
30	CH3-CH(CH3)-CN							93.31	93.43	156.2		1 point			
31	CH(O)-CH2-CH2-CH3	103.36	0.1	Chao_86 rcmd	101.8		eq. 39 Chao used CH2 + propanal	94.31	93.73	164.1	0.84	av. of 2 points			
32	CH3-C(O)-CH2-CH3	101.68	0.14	Chao_86 rcmd				98.33	97.89	158.6	0.4	av. of 9 points			
33	CH(O)-CH(CH3)-CH3							97.09	96.86				156.1		eq. 43
34	CH3-CH2-CHCH2	85.56		TRC_97 rcmd				82.15	82.19	128.96		1 point. Another value of 118 is doubtful			
35	CH3-C(=CH2)-CH3	88.09		TRC_97 rcmd				84.69	84.13				130.2		eq. 5
36	CH3-CH2-CH2-C(O)-NH2							116.76	116.49				158.1	1.7	av. over eqs. 50.1 - 50.3
37	CH3-CH(CH3)-C(O)-NH2							119.44	118.58	148.1		1 point			
38	CH3-CH2-CH2-CH2-OH	108.03	0.25	TRC_97 rcmd				104.59	104.58	176.9	0.5	av. of 8 points			
39	CH3-CH(CH3)-CH2-OH				108.6		eq. 22	106.98	107.13	181.4	0.57	av. of 5 points			
40	CH3-CH2-CH(CH3)-OH	112.74	0.17	TRC_97 rcmd				107.37	107.69	197.2	0.54	av. of 3 points			

No.	Compound	Cp (NIST)	Error	Comment	Cp(est)	Error	Comment	Cp(B3LYP)	Cp(M06-2X)	Cp(NIST)liquid	Error	Comment	Cp(est)liquid	Error	Comment
41	CH3-C(CH3)2-OH	113.63	0.21	TRC_97 rcmd				111.76	111.47	218.2	5.78	av. of 5 points			
42	CH3-CH2-CH2-O-CH3				108.1	0.9	av. over eqs. 24.1 - 24.4	102.79	103.30	165.4	0.07	av. of 2 points			
43	CH3-CH2-O-CH2-CH3	119.46	0.15	Chao_86 rcmd, bad value?	111.0		My estimation. Stull add.scheme gives 112.5	103.05	102.48	172.5		1 point. Too high?	165.4		Av. Of PrOEt-PrOMe and EtOMe-MeOMe + EtOMe
44	CH3-CH(CH3)-O-CH3				112.1		eq. 26	105.95	105.40	161.9		1 point			
45	HO-CH2-CH2-CH2-CH2-OH				120.6	2.0	av. over eqs. 27.1 - 27.5	119.34	119.71	200.1		1 point			
46	CH3-O-CH2-CH2-O-CH3				121.9	1.1	av. over eqs. 28.1 - 28.5	115.78	115.64	192.2	1.53	av. of 2 points			
47	CH3-CH2-CH2-CH3	98.49		Chen_75 rcmd				89.85	89.45				137.2		Regression for linear alkanes: 19.80 +- 1.1 + 29.36 +- 0.14 x n
48	CH3-CH(CH3)-CH3	96.65		Chen_75 rcmd				92.18	92.71				133.5	1.5	av. over 4 eqs. 7, 14.1, 14.2, 15.2; 129.7 (260 K)
49	CH3-CH2-CH2-CH2-NH2							107.63	108.17	188		1 point			
50	CH3-CH(CH3)-CH2-NH2							110.25	110.38	194		1 point			
51	CH3-CH2-CH(CH3)-NH2							110.33	110.47				193.7		eq. 49
52	CH3-CH2-CH2-CCH	102.56		TRC_97 rcmd				100.02	98.66						
53	CH3-CH2-CC-CH3	99.59		TRC_97 rcmd				100.09	99.37						
54	CH3-CH2-CH2-CH2-CN							111.11	111.48				166.1	2.9	av. over eqs. 52.1 - 52.5
55	CH3-CH2-CH(CH3)-CN							113.68	113.53				185.5		eq. 53
56	CH3-C(O)-CH2-CH2-CH3				123.7	1.9	av. over eqs. 41.1 - 41.5	118.45	118.32	184.7	0.54	av. of 5 points			
57	CH3-CH2-C(O)-CH2-CH3				126.4	1.7	av. over eqs. 42.1 - 42.4	118.42	117.49	194	4.31	av. of 6 points			
58	CH3-C(O)-CH(CH3)-CH3							120.61	120.22	180	0.07	av. of 2 points			
59	CH3-CH2-CH2-CHCH2	108.2		TRC_97 rcmd				102.33	102.06	154.7	0.67	av. of 3 points			
60	CH3-CH2-C(=CH2)-CH3	109.96		McCullough_59 rcmd				104.41	104.82	157.2	0.08	av. of 2 points			
61	CH3-CH2-CH2-CH2-CH2-OH				130.3	2.1	av. over eqs. 29.1 - 29.6	124.72	124.70	208.2	0.68	av. of 8 points			
62	CH3-CH(CH3)-CH2-CH2-OH				132.3	1.7	av. over eqs. 30.1 - 30.3	127.11	127.45	209.52		295.5 K			
63	CH3-CH2-CH(CH3)-CH2-OH				132.6	1.5	av. over eqs. 31.1 - 31.3	126.93	126.99				203.9	6.0	av. over eqs. 31.1 - 31.3
64	CH3-CH2-CH2-CH(CH3)-OH				135.0	1.0	av. over eqs. 32.1 - 32.3	127.38	127.06				225.0	4.0	av. over eqs. 32.1 - 32.3
65	CH3-CH(CH3)-CH(CH3)-OH				133.2		eq. 33	129.99	129.81	245.9		1 point			
66	CH3-CH2-CH2-CH2-O-CH3				130.4	2.2	av. over eqs. 34.1 - 34.7	123.07	123.29	192.7	0.26	av. of 3 points			
67	CH3-CH2-CH2-O-CH2-CH3				129.7	1.3	av. over eqs. 35.1 - 35.6	123.39	123.14	197.4	0.21	av. of 2 points			
68	CH3-C(CH3)2-O-CH3				136.4		eq. 36	130.87	129.72	187.8	0.25	av. of 3 points			
69	CH3-CH2-CH2-CH2-CH3	120.0	0.1	Scott_74 rcmd				110.01	109.41	167.9	1	av. of 2 points			
70	CH3-CH(CH3)-CH2-CH3	118.9	0.4	Scott_74 rcmd				112.50	112.58	164.7	0.25	av. of 2 points			
71	CH3-C(CH3)2-CH3	120.83	0.25	Scott_74 rcmd				114.59	113.00				159.0		eq. 16; 153.1 (260 K)
72	CH3-CH2-CH2-CH2-CCH	125.95		TRC_97 rcmd				120.10	118.59						
73	CH3-CH2-CH2-CC-CH3	119.65		TRC_97 rcmd				120.56	119.60						
74	CH3-CH2-CC-CH2-CH3				120.6	0.4	av. over eqs. 10.1 - 10.4	119.44	118.97						
75	CH3-CH2-CH2-CH2-CHCH2				130.5	2.0	av. over eqs. 11.1 - 11.5	122.64	122.77	183	0.37	av. of 2 points			
76	CH3-CH2-C(=CH2)-CH2-CH3				131.6	0.9	av. over eqs. 12.1 - 12.4	124.17	123.60				183.9	0.6	av. over eqs. 12.1 - 12.4
77	CH3-CH2-CH2-CH2-CH2-CH3	142.6	0.2	Scott_74 rcmd				130.33	131.09	196	1.15	av. of 18 points			
78	CH3-CH(CH3)-CH2-CH2-CH3	142.2	0.2	Scott_74 rcmd				132.50	132.54	193.9	0.16	av. of 6 points			
79	CH3-CH2-CH(CH3)-CH2-CH3	140.1	0.4	Scott_74 rcmd				132.83	133.06	190.9	0.18	av. of 6 points			
80	CH3-C(CH3)2-CH2-CH3	141.5	0.3	Scott_74 rcmd				136.19	136.63	190.2	1.32	av. of 6 points			

All heat capacities are given in J/mol·K

Table S2. Complete sets of HDRs. *Homodesmotic reactions of 2 level are highlighted by yellow, HDR of 3 level – by green. Heat capacities are corresponded to the B3LYP-calculated values.*

HDR No.	ΔC_p	Phase	HDR reagents and their *CP*						HDR products and their CP				
Hydrocarbons													
1			CH3-CH2-CH2-CH3	+	CH3-CH3			-->	CH3-CH2-CH3	+	CH3-CH2-CH3		
	-0.54	gas	89.85		50.11				69.71		69.71		
2			CH3-CH2-CH2-CCH	+	CH3-CH3			-->	CH3-CH2-CH3	+	CH3-CH2-CCH		
	-0.76	gas	100.02		50.11				69.71		79.65		
3			CH3-CH2-CC-CH3	+	CH3-CCH			-->	CH3-CC-CH3	+	CH3-CH2-CCH		
	-0.13	gas	100.09		60.47				80.78		79.65		
4			CH3-CH2-CH2-CHCH2	+	CH3-CH3			-->	CH3-CH2-CH3	+	CH3-CH2-CHCH2		
	-0.58	gas	102.33		50.11				69.71		82.15		
5			CH3-CH2-C(=CH2)-CH3	+	CH3-CHCH2			-->	CH3-CH2-CHCH2	+	CH3-C(=CH2)-CH3		
	0.10	gas	104.41		62.32				82.15		84.69		
6			CH3-CH2-CH2-CH2-CH3	+	CH3-CH3			-->	CH3-CH2-CH3	+	CH3-CH2-CH2-CH3		
6.1	-0.56	gas	110.01		50.11				69.71		89.85		
			CH3-CH2-CH2-CH2-CH3	+	CH3-CH3	+	CH3-CH3	-->	CH3-CH2-CH3	+	CH3-CH2-CH3	+	CH3-CH2-CH3
6.2	-1.10	gas	110.01		50.11		50.11		69.71		69.71		69.71
			CH3-CH2-CH2-CH2-CH3	+	CH3-CH2-CH3			-->	CH3-CH2-CH2-CH3	+	CH3-CH2-CH2-CH3		
6.3	-0.03	gas	110.01		69.71				89.85		89.85		
7			CH3-CH(CH3)-CH2-CH3	+	CH3-CH2-CH3			-->	CH3-CH(CH3)-CH3	+	CH3-CH2-CH2-CH3		
	-0.19	gas	112.50		69.71				92.18		89.85		
8			CH3-CH2-CH2-CH2-CCH	+	CH3-CH3			-->	CH3-CH2-CH3	+	CH3-CH2-CH2-CCH		
8.1	-0.48	gas	120.10		50.11				69.71		100.02		
			CH3-CH2-CH2-CH2-CCH	+	CH3-CH3			-->	CH3-CH2-CH2-CH3	+	CH3-CH2-CCH		
8.2	-0.71	gas	120.10		50.11				89.85		79.65		
			CH3-CH2-CH2-CH2-CCH	+	CH3-CH3	+	CH3-CH3	-->	CH3-CH2-CH3	+	CH3-CH2-CH3	+	CH3-CH2-CCH
8.3	-1.24	gas	120.10		50.11		50.11		69.71		69.71		79.65
			CH3-CH2-CH2-CH2-CCH	+	CH3-CH2-CH3			-->	CH3-CH2-CH2-CH3	+	CH3-CH2-CH2-CCH		
8.4	0.05	gas	120.10		69.71				89.85		100.02		
			CH3-CH2-CH2-CH2-CCH	+	CH3-CH2-CCH			-->	CH3-CH2-CH2-CCH	+	CH3-CH2-CH2-CCH		
8.5	0.28	gas	120.10		79.65				100.02		100.02		

HDR No.	ΔC_p	Phase	HDR reagents and their *CP*						HDR products and their CP				
9			CH3-CH2-CH2-CC-CH3	+	CH3-CH3			-->	CH3-CH2-CH3	+	CH3-CH2-CC-CH3		
9.1	-0.87	gas	120.56		50.11				69.71		100.09		
			CH3-CH2-CH2-CC-CH3	+	CH3-CCH			-->	CH3-CH2-CH2-CCH	+	CH3-CC-CH3		
9.2	-0.24	gas	120.56		60.47				100.02		80.78		
			CH3-CH2-CH2-CC-CH3	+	CH3-CH3	+	CH3-CCH	-->	CH3-CH2-CH3	+	CH3-CH2-CCH	+	CH3-CC-CH3
9.3	-1.00	gas	120.56		50.11		60.47		69.71		79.65		80.78
			CH3-CH2-CH2-CC-CH3	+	CH3-CH2-CCH			-->	CH3-CH2-CH2-CCH	+	CH3-CH2-CC-CH3		
9.4	-0.11	gas	120.56		79.65				100.02		100.09		
			CH3-CH2-CH2-CC-CH3	+	CH3-CC-CH3	+		-->	CH3-CH2-CC-CH3	+	CH3-CH2-CC-CH3		
9.5	-1.17	gas	120.56		80.78				100.09		100.09		
10			CH3-CH2-CC-CH2-CH3	+	CH3-CCH			-->	CH3-CH2-CCH	+	CH3-CH2-CC-CH3		
10.1	-0.18	gas	119.44		60.47				79.65		100.09		
			CH3-CH2-CC-CH2-CH3	+	CH3-CCH	+	CH3-CCH	-->	CH3-CH2-CCH	+	CH3-CH2-CCH	+	CH3-CC-CH3
10.2	-0.31	gas	119.44		60.47		60.47		79.65		79.65		80.78
			CH3-CH2-CC-CH2-CH3	+	CH3-CH2-CCH			-->	CH3-CH2-CC-CH3	+	CH3-CH2-CH2-CCH		
10.3	1.01	gas	119.44		79.65				100.09		100.02		
			CH3-CH2-CC-CH2-CH3	+	CH3-CC-CH3			-->	CH3-CH2-CC-CH3	+	CH3-CH2-CC-CH3		
10.4	-0.05	gas	119.44		80.78				100.09		100.09		
11			CH3-CH2-CH2-CH2-CHCH2	+	CH3-CH3			-->	CH3-CH2-CH3	+	CH3-CH2-CH2-CHCH2		
11.1	-0.70	gas	122.64		50.11				69.71		102.33		
			CH3-CH2-CH2-CH2-CHCH2	+	CH3-CH3			-->	CH3-CH2-CH2-CH3	+	CH3-CH2-CHCH2		
11.2	-0.75	gas	122.64		50.11				89.85		82.15		
			CH3-CH2-CH2-CH2-CHCH2	+	CH3-CH3	+	CH3-CH3	-->	CH3-CH2-CH3	+	CH3-CH2-CH3	+	CH3-CH2-CHCH2
11.3	-1.28	gas	122.64		50.11		50.11		69.71		69.71		82.15
			CH3-CH2-CH2-CH2-CHCH2	+	CH3-CH2-CH3			-->	CH3-CH2-CH2-CH3	+	CH3-CH2-CH2-CHCH2		
11.4	-0.17	gas	122.64		69.71				89.85		102.33		
			CH3-CH2-CH2-CH2-CHCH2	+	CH3-CH2-CHCH2			-->	CH3-CH2-CH2-CHCH2	+	CH3-CH2-CH2-CHCH2		
11.5	-0.12	gas	122.64		82.15				102.33		102.33		
12			CH3-CH2-C(=CH2)-CH2-CH3	+	CH3-CHCH2			-->	CH3-CH2-CHCH2	+	CH3-CH2-C(=CH2)-CH3		
12.1	0.07	gas	124.17		62.32				82.15		104.41		
			CH3-CH2-C(=CH2)-CH2-CH3	+	CH3-CHCH2	+	CH3-CHCH2	-->	CH3-CH2-CHCH2	+	CH3-CH2-CHCH2	+	CH3-C(=CH2)-CH3
12.2	0.17	gas	124.17		62.32		62.32		82.15		82.15		84.69
			CH3-CH2-C(=CH2)-CH2-CH3	+	CH3-CH2-CHCH2			-->	CH3-CH2-CH2-CHCH2	+	CH3-CH2-C(=CH2)-CH3		
12.3	0.42	gas	124.17		82.15				102.33		104.41		
			CH3-CH2-C(=CH2)-CH2-CH3	+	CH3-C(=CH2)-CH3			-->	CH3-CH2-C(=CH2)-CH3	+	CH3-CH2-C(=CH2)-CH3		
12.4	-0.04	gas	124.17		84.69				104.41		104.41		

HDR No.	ΔC_P	Phase	**HDR reagents and their *CP***								**HDR products and their CP**						
13			CH3-CH2-CH2-CH2-CH2-CH3	+	CH3-CH3					-->	CH3-CH2-CH3	+	CH3-CH2-CH2-CH2-CH3				
13.1	-0.72	gas	130.33		50.11						69.71		110.01				
			CH3-CH2-CH2-CH2-CH2-CH3	+	CH3-CH3					-->	CH3-CH2-CH2-CH3	+	CH3-CH2-CH2-CH3				
13.2	-0.74	gas	130.33		50.11						89.85		89.85				
			CH3-CH2-CH2-CH2-CH2-CH3	+	CH3-CH3	+	CH3-CH3			-->	CH3-CH2-CH3	+	CH3-CH2-CH3	+	CH3-CH2-CH2-CH3		
13.3	-1.28	gas	130.33		50.11		50.11				69.71		69.71		89.85		
			CH3-CH2-CH2-CH2-CH2-CH3	+	CH3-CH3	+	CH3-CH3	+	CH3-CH3	-->	CH3-CH2-CH3	+	CH3-CH2-CH3	+	CH3-CH2-CH3	+	CH3-CH2-CH3
13.4	-1.82	gas	130.33		50.11		50.11		50.11		69.71		69.71		69.71		69.71
			CH3-CH2-CH2-CH2-CH2-CH3	+	CH3-CH2-CH3					-->	CH3-CH2-CH2-CH3	+	CH3-CH2-CH2-CH2-CH3				
13.5	-0.18	gas	130.33		69.71						89.85		110.01				
			CH3-CH2-CH2-CH2-CH2-CH3	+	CH3-CH2-CH2-CH3					-->	CH3-CH2-CH2-CH2-CH3	+	CH3-CH2-CH2-CH2-CH3				
13.6	-0.15	gas	130.33		89.85						110.01		110.01				
14			CH3-CH(CH3)-CH2-CH2-CH3	+	CH3-CH3					-->	CH3-CH2-CH3	+	CH3-CH(CH3)-CH2-CH3				
14.1	-0.40	gas	132.50		50.11						69.71		112.50				
			CH3-CH(CH3)-CH2-CH2-CH3	+	CH3-CH2-CH3					-->	CH3-CH2-CH2-CH2-CH3	+	CH3-CH(CH3)-CH3				
14.2	-0.02	gas	132.50		69.71						110.01		92.18				
			CH3-CH(CH3)-CH2-CH2-CH3	+	CH3-CH3	+	CH3-CH2-CH3			-->	CH3-CH2-CH3	+	CH3-CH(CH3)-CH3	+	CH3-CH2-CH2-CH3		
14.3	-0.59	gas	132.50		50.11		69.71				69.71		92.18		89.85		
15			CH3-CH2-CH(CH3)-CH2-CH3	+	CH3-CH2-CH3					-->	CH3-CH2-CH2-CH3	+	CH3-CH(CH3)-CH2-CH3				
15.1	-0.19	gas	132.83		69.71						89.85		112.50				
			CH3-CH2-CH(CH3)-CH2-CH3	+	CH3-CH2-CH3	+	CH3-CH2-CH3			-->	CH3-CH2-CH2-CH3	+	CH3-CH2-CH2-CH3	+	CH3-CH(CH3)-CH3		
15.2	-0.38	gas	132.83		69.71		69.71				89.85		89.85		92.18		
16			CH3-C(CH3)2-CH2-CH3	+	CH3-CH(CH3)-CH3					-->	CH3-CH(CH3)-CH2-CH3	+	CH3-C(CH3)2-CH3				
	-1.27	gas	136.19		92.18						112.50		114.59				
Alcohols and ethers																	
17			HO-CH2-CH2-OH	+	CH3-CH3					-->	CH3-CH2-OH	+	CH3-CH2-OH				
	2.26	gas	76.12		50.11						64.25		64.25				
18			CH3-CH2-CH2-OH	+	CH3-CH3					-->	CH3-CH2-CH3	+	CH3-CH2-OH				
	-0.58	gas	84.43		50.11						69.71		64.25				
19			CH3-CH2-O-CH3	+	CH3-OH					-->	CH3-O-CH3	+	CH3-CH2-OH				
	-0.86	gas	82.53		44.61						62.03		64.25				

HDR No.	ΔC_p	Phase	HDR reagents and their *CP*								HDR products and their CP						
20			CH3-O-CH2-CH2-OH	+	CH3-CH3					-->	CH3-CH2-OH	+	CH3-CH2-O-CH3				
20.1	1.22	gas	95.45		50.11						64.25		82.53				
			CH3-O-CH2-CH2-OH	+	CH3-OH					-->	CH3-O-CH3	+	HO-CH2-CH2-OH				
20.2	-1.91	gas	95.45		44.61						62.03		76.12				
			CH3-O-CH2-CH2-OH	+	CH3-CH3	+	CH3-OH			-->	CH3-CH2-OH	+	CH3-CH2-OH	+	CH3-O-CH3		
20.3	0.36	gas	95.45		50.11		44.61				64.25		64.25		62.03		
			CH3-O-CH2-CH2-OH	+	CH3-CH2-OH					-->	CH3-CH2-O-CH3	+	HO-CH2-CH2-OH				
20.4	-1.05	gas	95.45		64.25						82.53		76.12				
21			CH3-CH2-CH2-CH2-OH	+	CH3-CH3					-->	CH3-CH2-CH3	+	CH3-CH2-CH2-OH				
21.1	-0.56	gas	104.59		50.11						69.71		84.43				
			CH3-CH2-CH2-CH2-OH	+	CH3-CH3					-->	CH3-CH2-CH2-CH3	+	CH3-CH2-OH				
21.2	-0.60	gas	104.59		50.11						89.85		64.25				
			CH3-CH2-CH2-CH2-OH	+	CH3-CH3	+	CH3-CH3			-->	CH3-CH2-CH3	+	CH3-CH2-CH3	+	CH3-CH2-OH		
21.3	-1.14	gas	104.59		50.11		50.11				69.71		69.71		64.25		
22			CH3-CH(CH3)-CH2-OH	+	CH3-CH2-CH3					-->	CH3-CH2-CH2-OH	+	CH3-CH(CH3)-CH3				
	-0.08	gas	106.98		69.71						84.43		92.18				
23			CH3-CH2-CH(CH3)-OH	+	CH3-CH2-CH3					-->	CH3-CH2-CH2-CH3	+	CH3-CH(CH3)-OH				
	-0.22	gas	107.37		69.71						89.85		87.02				
24			CH3-CH2-CH2-O-CH3	+	CH3-CH3					-->	CH3-CH2-CH3	+	CH3-CH2-O-CH3				
24.1	-0.66	gas	102.79		50.11						69.71		82.53				
			CH3-CH2-CH2-O-CH3	+	CH3-OH					-->	CH3-O-CH3	+	CH3-CH2-CH2-OH				
24.2	-0.94	gas	102.79		44.61						62.03		84.43				
			CH3-CH2-CH2-O-CH3	+	CH3-CH3	+	CH3-OH			-->	CH3-CH2-CH3	+	CH3-CH2-OH	+	CH3-O-CH3		
24.3	-1.52	gas	102.79		50.11		44.61				69.71		64.25		62.03		
			CH3-CH2-CH2-O-CH3	+	CH3-CH2-OH					-->	CH3-CH2-O-CH3	+	CH3-CH2-CH2-OH				
24.4	-0.08	gas	102.79		64.25						82.53		84.43				
25			CH3-CH2-O-CH2-CH3	+	CH3-OH					-->	CH3-CH2-OH	+	CH3-CH2-O-CH3				
25.1	-0.88	gas	103.05		44.61						64.25		82.53				
			CH3-CH2-O-CH2-CH3	+	CH3-OH	+	CH3-OH			-->	CH3-CH2-OH	+	CH3-CH2-OH	+	CH3-O-CH3		
25.2	-1.74	gas	103.05		44.61		44.61				64.25		64.25		62.03		
			CH3-CH2-O-CH2-CH3	+	CH3-O-CH3					-->	CH3-CH2-O-CH3	+	CH3-CH2-O-CH3				
25.3	-0.02	gas	103.05		62.03						82.53		82.53				
26			CH3-CH(CH3)-O-CH3	+	CH3-CH2-OH					-->	CH3-CH(CH3)-OH	+	CH3-CH2-O-CH3				
	-0.64	gas	105.95		64.25						87.02		82.53				

HDR No.	ΔC_P	Phase	HDR reagents and their *CP*								HDR products and their CP						
27			HO-CH2-CH2-CH2-CH2-OH	+	CH3-CH3					-->	CH3-CH2-OH	+	CH3-CH2-CH2-CH2-OH				
27.1	-0.62	gas	119.34		50.11						64.25		104.59				
			HO-CH2-CH2-CH2-CH2-OH	+	CH3-CH3					-->	CH3-CH2-CH2-OH	+	CH3-CH2-CH2-OH				
27.2	-0.59	gas	119.34		50.11						84.43		84.43				
			HO-CH2-CH2-CH2-CH2-OH	+	CH3-CH3	+	CH3-CH3			-->	CH3-CH2-OH	+	CH3-CH2-OH	+	CH3-CH2-CH2-CH3		
27.3	-1.22	gas	119.34		50.11		50.11				64.25		64.25		89.85		
			HO-CH2-CH2-CH2-CH2-OH	+	CH3-CH3	+	CH3-CH3			-->	CH3-CH2-OH	+	CH3-CH2-CH2-OH	+	CH3-CH2-CH3		
27.4	-1.17	gas	119.34		50.11		50.11				64.25		84.43		69.71		
			HO-CH2-CH2-CH2-CH2-OH	+	CH3-CH3	+	CH3-CH3	+	CH3-CH3	-->	CH3-CH2-CH3	+	CH3-CH2-CH3	+	CH3-CH2-OH	+	CH3-CH2-OH
27.5	-1.75	gas	119.34		50.11		50.11		50.11		69.71		69.71		64.25		64.25
28			CH3-O-CH2-CH2-O-CH3	+	CH3-CH3					-->	CH3-CH2-O-CH3	+	CH3-CH2-O-CH3				
28.1	-0.83	gas	115.78		50.11						82.53		82.53				
			CH3-O-CH2-CH2-O-CH3	+	CH3-OH					-->	CH3-O-CH3	+	CH3-O-CH2-CH2-OH				
28.2	-2.91	gas	115.78		44.61						62.03		95.45				
			CH3-O-CH2-CH2-O-CH3	+	CH3-CH3	+	CH3-OH			-->	CH3-O-CH3	+	CH3-CH2-OH	+	CH3-CH2-O-CH3		
28.3	-1.69	gas	115.78		50.11		44.61				62.03		64.25		82.53		
			CH3-O-CH2-CH2-O-CH3	+	CH3-OH	+	CH3-OH			-->	HO-CH2-CH2-OH	+	CH3-O-CH3	+	CH3-O-CH3		
28.4	-4.82	gas	115.78		44.61		44.61				76.12		62.03		62.03		
			CH3-O-CH2-CH2-O-CH3	+	CH3-CH3	+	CH3-OH	+	CH3-OH	-->	CH3-O-CH3	+	CH3-O-CH3	+	CH3-CH2-OH	+	CH3-CH2-OH
28.5	-2.55	gas	115.78		50.11		44.61		44.61		62.03		62.03		64.25		64.25
29			CH3-CH2-CH2-CH2-CH2-OH	+	CH3-CH3					-->	CH3-CH2-OH	+	CH3-CH2-CH2-CH2-CH3				
29.1	-0.57	gas	124.72		50.11						64.25		110.01				
			CH3-CH2-CH2-CH2-CH2-OH	+	CH3-CH3					-->	CH3-CH2-CH2-OH	+	CH3-CH2-CH2-CH3				
29.2	-0.55	gas	124.72		50.11						84.43		89.85				
			CH3-CH2-CH2-CH2-CH2-OH	+	CH3-CH3					-->	CH3-CH2-CH2-CH2-OH	+	CH3-CH2-CH3				
29.3	-0.53	gas	124.72		50.11						104.59		69.71				
			CH3-CH2-CH2-CH2-CH2-OH	+	CH3-CH3	+	CH3-CH3			-->	CH3-CH2-OH	+	CH3-CH2-CH3	+	CH3-CH2-CH2-CH3		
29.4	-1.13	gas	124.72		50.11		50.11				64.25		69.71		89.85		
			CH3-CH2-CH2-CH2-CH2-OH	+	CH3-CH3	+	CH3-CH3			-->	CH3-CH2-CH2-OH	+	CH3-CH2-CH3	+	CH3-CH2-CH3		
29.5	-1.09	gas	124.72		50.11		50.11				84.43		69.71		69.71		
			CH3-CH2-CH2-CH2-CH2-OH	+	CH3-CH3	+	CH3-CH3	+	CH3-CH3	-->	CH3-CH2-CH3	+	CH3-CH2-CH3	+	CH3-CH2-CH3	+	CH3-CH2-OH
29.6	-1.67	gas	124.72		50.11		50.11		50.11		69.71		69.71		69.71		64.25
30			CH3-CH(CH3)-CH2-CH2-OH	+	CH3-CH3					-->	CH3-CH2-OH	+	CH3-CH(CH3)-CH2-CH3				
30.1	-0.46	gas	127.11		50.11						64.25		112.50				
			CH3-CH(CH3)-CH2-CH2-OH	+	CH3-CH2-CH3					-->	CH3-CH2-CH2-CH2-OH	+	CH3-CH(CH3)-CH3				
30.2	-0.05	gas	127.11		69.71						104.59		92.18				
			CH3-CH(CH3)-CH2-CH2-OH	+	CH3-CH3	+	CH3-CH2-CH3			-->	CH3-CH2-OH	+	CH3-CH(CH3)-CH3	+	CH3-CH2-CH2-CH3		
30.3	-0.65	gas	127.11		50.11		69.71				64.25		92.18		89.85		

HDR No.	ΔC_P	Phase	HDR reagents and their *CP*								HDR products and their CP						
31			CH3-CH2-CH(CH3)-CH2-OH	+	CH3-CH2-CH3					-->	CH3-CH2-CH2-OH	+	CH3-CH(CH3)-CH2-CH3				
31.1	0.29	gas	126.93		69.71						84.43		112.50				
			CH3-CH2-CH(CH3)-CH2-OH	+	CH3-CH2-CH3					-->	CH3-CH2-CH2-CH3	+	CH3-CH(CH3)-CH2-OH				
31.2	0.18	gas	126.93		69.71						89.85		106.98				
			CH3-CH2-CH(CH3)-CH2-OH	+	CH3-CH2-CH3	+	CH3-CH2-CH3			-->	CH3-CH2-CH2-CH3	+	CH3-CH(CH3)-CH3	+	CH3-CH2-CH2-OH		
31.3	0.10	gas	126.93		69.71		69.71				89.85		92.18		84.43		
32			CH3-CH2-CH2-CH(CH3)-OH	+	CH3-CH3					-->	CH3-CH2-CH3	+	CH3-CH2-CH(CH3)-OH				
32.1	-0.40	gas	127.38		50.11						69.71		107.37				
			CH3-CH2-CH2-CH(CH3)-OH	+	CH3-CH2-CH3					-->	CH3-CH2-CH2-CH2-CH3	+	CH3-CH(CH3)-OH				
32.2	-0.05	gas	127.38		69.71						110.01		87.02				
			CH3-CH2-CH2-CH(CH3)-OH	+	CH3-CH3	+	CH3-CH2-CH3			-->	CH3-CH2-CH2-CH3	+	CH3-CH2-CH3	+	CH3-CH(CH3)-OH		
32.3	-0.62	gas	127.38		50.11		69.71				89.85		69.71		87.02		
33			CH3-CH(CH3)-CH(CH3)-OH	+	CH3-CH2-CH2-CH3					-->	CH3-CH2-CH(CH3)-OH	+	CH3-CH(CH3)-CH2-CH3				
	0.04	gas	129.99		89.85						107.37		112.50				
34			CH3-CH2-CH2-CH2-O-CH3	+	CH3-CH3					-->	CH3-CH2-CH2-CH3	+	CH3-CH2-O-CH3				
34.1	-0.80	gas	123.07		50.11						89.85		82.53				
			CH3-CH2-CH2-CH2-O-CH3	+	CH3-CH3					-->	CH3-CH2-CH3	+	CH3-CH2-CH2-O-CH3				
34.2	-0.68	gas	123.07		50.11						69.71		102.79				
			CH3-CH2-CH2-CH2-O-CH3	+	CH3-OH					-->	CH3-O-CH3	+	CH3-CH2-CH2-CH2-OH				
34.3	-1.06	gas	123.07		44.61						62.03		104.59				
			CH3-CH2-CH2-CH2-O-CH3	+	CH3-CH3	+	CH3-CH3			-->	CH3-CH2-CH3	+	CH3-CH2-CH3	+	CH3-CH2-O-CH3		
34.4	-1.33	gas	123.07		50.11		50.11				69.71		69.71		82.53		
			CH3-CH2-CH2-CH2-O-CH3	+	CH3-CH3	+	CH3-OH			-->	CH3-O-CH3	+	CH3-CH2-CH2-CH3	+	CH3-CH2-OH		
34.5	-1.66	gas	123.07		50.11		44.61				62.03		89.85		64.25		
			CH3-CH2-CH2-CH2-O-CH3	+	CH3-CH3	+	CH3-OH			-->	CH3-O-CH3	+	CH3-CH2-CH3	+	CH3-CH2-CH2-OH		
34.6	-1.62	gas	123.07		50.11		44.61				62.03		69.71		84.43		
			CH3-CH2-CH2-CH2-O-CH3	+	CH3-CH3	+	CH3-CH3	+	CH3-OH	-->	CH3-O-CH3	+	CH3-CH2-OH	+	CH3-CH2-CH3	+	CH3-CH2-CH3
34.7	-2.20	gas	123.07		50.11		50.11		44.61		62.03		64.25		69.71		69.71
35			CH3-CH2-CH2-O-CH2-CH3	+	CH3-CH3					-->	CH3-CH2-CH3	+	CH3-CH2-O-CH2-CH3				
35.1	-0.73	gas	123.39		50.11						69.71		103.05				
			CH3-CH2-CH2-O-CH2-CH3	+	CH3-OH					-->	CH3-CH2-OH	+	CH3-CH2-CH2-O-CH3				
35.2	-0.96	gas	123.39		44.61						64.25		102.79				
			CH3-CH2-CH2-O-CH2-CH3	+	CH3-OH					-->	CH3-CH2-CH2-OH	+	CH3-CH2-O-CH3				
35.3	-1.03	gas	123.39		44.61						84.43		82.53				
			CH3-CH2-CH2-O-CH2-CH3	+	CH3-CH3	+	CH3-OH			-->	CH3-CH2-CH3	+	CH3-CH2-OH	+	CH3-CH2-O-CH3		
35.4	-1.62	gas	123.39		50.11		44.61				69.71		64.25		82.53		
			CH3-CH2-CH2-O-CH2-CH3	+	CH3-OH	+	CH3-OH			-->	CH3-O-CH3	+	CH3-CH2-CH2-OH	+	CH3-CH2-OH		
35.5	-1.90	gas	123.39		44.61		44.61				62.03		84.43		64.25		
			CH3-CH2-CH2-O-CH2-CH3	+	CH3-CH3	+	CH3-OH	+	CH3-OH	-->	CH3-O-CH3	+	CH3-CH2-CH3	+	CH3-CH2-OH	+	CH3-CH2-OH
35.6	-2.48	gas	123.39		50.11		44.61		44.61		62.03		69.71		64.25		64.25

HDR No.	ΔC_P	Phase	HDR reagents and their *CP*								HDR products and their CP						
36			CH3-C(CH3)2-O-CH3	+	CH3-CH(CH3)-OH					-->	CH3-C(CH3)2-OH	+	CH3-CH(CH3)-O-CH3				
	-0.19	gas	130.87		87.02						111.76		105.95				
Carbonyl compounds																	
37			CH3-C(O)-O-CH3	+	CH(O)-OH					-->	CH3-C(O)-OH	+	CH(O)-O-CH3				
	-0.78	gas	89.35		44.19						67.80		64.97				
38			CH(O)-O-CH2-CH3	+	CH3-OH					-->	CH(O)-O-CH3	+	CH3-CH2-OH				
	-0.82	gas	85.42		44.61						64.97		64.25				
39			CH(O)-CH2-CH2-CH3	+	CH3-CH3					-->	CH(O)-CH2-CH3	+	CH3-CH2-CH3				
	-0.37	gas	94.31		50.11						74.34		69.71				
40			CH3-C(O)-CH2-CH3	+	CH(O)-CH3					-->	CH3-C(O)-CH3	+	CH(O)-CH2-CH3				
	-0.46	gas	98.33		54.96						78.49		74.34				
41			CH3-C(O)-CH2-CH2-CH3	+	CH(O)-CH3					-->	CH3-C(O)-CH3	+	CH(O)-CH2-CH2-CH3				
41.1	-0.62	gas	118.45		54.96						78.49		94.31				
			CH3-C(O)-CH2-CH2-CH3	+	CH3-CH3					-->	CH3-CH2-CH3	+	CH3-C(O)-CH2-CH3				
41.2	-0.52	gas	118.45		50.11						69.71		98.33				
			CH3-C(O)-CH2-CH2-CH3	+	CH3-CH3	+	CH(O)-CH3			-->	CH3-CH2-CH3	+	CH3-C(O)-CH3	+	CH(O)-CH2-CH3		
41.3	-0.98	gas	118.45		50.11		54.96				69.71		78.49		74.34		
			CH3-C(O)-CH2-CH2-CH3	+	CH3-CH2-CH3					-->	CH3-C(O)-CH2-CH3	+	CH3-CH2-CH2-CH3				
41.4	0.02	gas	118.45		69.71						98.33		89.85				
			CH3-C(O)-CH2-CH2-CH3	+	CH(O)-CH2-CH3					-->	CH(O)-CH2-CH2-CH3	+	CH3-C(O)-CH2-CH3				
41.5	-0.15	gas	118.45		74.34						94.31		98.33				
42			CH3-CH2-C(O)-CH2-CH3	+	CH(O)-CH3					-->	CH3-C(O)-CH2-CH3	+	CH(O)-CH2-CH3				
42.1	-0.71	gas	118.42		54.96						98.33		74.34				
			CH3-CH2-C(O)-CH2-CH3	+	CH(O)-CH3	+	CH(O)-CH3			-->	CH3-C(O)-CH3	+	CH(O)-CH2-CH3	+	CH(O)-CH2-CH3		
42.2	-1.18	gas	118.42		54.96		54.96				78.49		74.34		74.34		
			CH3-CH2-C(O)-CH2-CH3	+	CH(O)-CH2-CH3					-->	CH(O)-CH2-CH2-CH3	+	CH3-C(O)-CH2-CH3				
42.3	-0.12	gas	118.42		74.34						94.31		98.33				
			CH3-CH2-C(O)-CH2-CH3	+	CH3-C(O)-CH3					-->	CH3-C(O)-CH2-CH3	+	CH3-C(O)-CH2-CH3				
42.4	-0.25	gas	118.42		78.49						98.33		98.33				
43			CH3-C(O)-CH(CH3)-CH3	+	CH(O)-CH2-CH3					-->	CH3-C(O)-CH2-CH3	+	CH(O)-CH(CH3)-CH3				
	0.48	gas	120.61		74.34						98.33		97.09				
N-containing compounds																	
44			CH3-CH2-CH2-NH2	+	CH3-CH3					-->	CH3-CH2-CH3	+	CH3-CH2-NH2				
	-0.38	gas	87.37		50.11						69.71		67.38				
45			CH3-CH2-C(O)-NH2	+	CH(O)-CH3					-->	CH(O)-CH2-CH3	+	CH3-C(O)-NH2				
	-0.21	gas	96.74		54.96						74.34		77.15				

HDR No.	ΔC_P	Phase	HDR reagents and their *CP*								HDR products and their CP						
46			CH3-CH2-CH2-CN	+	CH3-CH3					-->	CH3-CH2-CH3	+	CH3-CH2-CN				
	-0.58	gas	90.82		50.11						69.71		70.64				
47			CH3-CH2-CH2-CH2-NH2	+	CH3-CH3					-->	CH3-CH2-CH3	+	CH3-CH2-CH2-NH2				
47.1	-0.65	gas	107.63		50.11						69.71		87.37				
			CH3-CH2-CH2-CH2-NH2	+	CH3-CH3					-->	CH3-CH2-CH2-CH3	+	CH3-CH2-NH2				
47.2	-0.50	gas	107.63		50.11						89.85		67.38				
			CH3-CH2-CH2-CH2-NH2	+	CH3-CH3	+	CH3-CH3			-->	CH3-CH2-CH3	+	CH3-CH2-NH2	+	CH3-CH2-CH3		
47.3	-1.04	gas	107.63		50.11		50.11				69.71		67.38		69.71		
			CH3-CH2-CH2-CH2-NH2	+	CH3-CH2-CH3					-->	CH3-CH2-CH2-CH3	+	CH3-CH2-CH2-NH2				
47.4	-0.12	gas	107.63		69.71						89.85		87.37				
			CH3-CH2-CH2-CH2-NH2	+	CH3-CH2-NH2					-->	CH3-CH2-CH2-NH2	+	CH3-CH2-CH2-NH2				
47.5	-0.27	gas	107.63		67.38						87.37		87.37				
48			CH3-CH(CH3)-CH2-NH2	+	CH3-CH2-CH3					-->	CH3-CH(CH3)-CH3	+	CH3-CH2-CH2-NH2				
	-0.41	gas	110.25		69.71						92.18		87.37				
49			CH3-CH2-CH(CH3)-NH2	+	CH3-CH2-CH3					-->	CH3-CH2-CH2-CH3	+	CH3-CH(CH3)-NH2				
	-0.18	gas	110.33		69.71						89.85		90.01				
50			CH3-CH2-CH2-C(O)-NH2	+	CH3-CH3					-->	CH3-CH2-CH3	+	CH3-CH2-C(O)-NH2				
50.1	-0.41	gas	116.76		50.11						69.71		96.74				
			CH3-CH2-CH2-C(O)-NH2	+	CH(O)-CH3					-->	CH(O)-CH2-CH2-CH3	+	CH3-C(O)-NH2				
50.2	-0.26	gas	116.76		54.96						94.31		77.15				
			CH3-CH2-CH2-C(O)-NH2	+	CH3-CH3	+	CH(O)-CH3			-->	CH3-CH2-CH3	+	CH(O)-CH2-CH3	+	CH3-C(O)-NH2		
50.3	-0.63	gas	116.76		50.11		54.96				69.71		74.34		77.15		
51			CH3-CH(CH3)-C(O)-NH2	+	CH(O)-CH2-CH3					-->	CH(O)-CH(CH3)-CH3	+	CH3-CH2-C(O)-NH2				
	0.06	gas	119.44		74.34						97.09		96.74				
52			CH3-CH2-CH2-CH2-CN	+	CH3-CH3					-->	CH3-CH2-CH3	+	CH3-CH2-CH2-CN				
52.1	-0.69	gas	111.11		50.11						69.71		90.82				
			CH3-CH2-CH2-CH2-CN	+	CH3-CH3					-->	CH3-CH2-CH2-CH3	+	CH3-CH2-CN				
52.2	-0.73	gas	111.11		50.11						89.85		70.64				
			CH3-CH2-CH2-CH2-CN	+	CH3-CH3	+	CH3-CH3			-->	CH3-CH2-CH3	+	CH3-CH2-CN	+	CH3-CH2-CH3		
52.3	-1.27	gas	111.11		50.11		50.11				69.71		70.64		69.71		
			CH3-CH2-CH2-CH2-CN	+	CH3-CH2-CH3					-->	CH3-CH2-CH2-CH3	+	CH3-CH2-CH2-CN				
52.4	-0.15	gas	111.11		69.71						89.85		90.82				
			CH3-CH2-CH2-CH2-CN	+	CH3-CH2-CN					-->	CH3-CH2-CH2-CN	+	CH3-CH2-CH2-CN				
52.5	-0.10	gas	111.11		70.64						90.82		90.82				
53			CH3-CH2-CH(CH3)-CN	+	CH3-CH2-CH3					-->	CH3-CH2-CH2-CH3	+	CH3-CH(CH3)-CN				
	-0.23	gas	113.68		69.71						89.85		93.31				

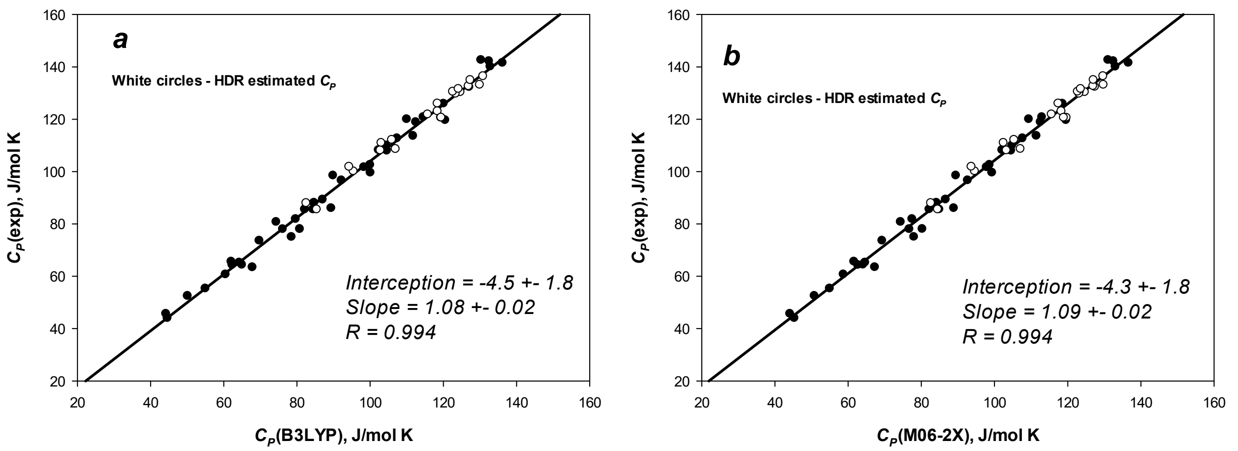

Figure S1. Direct *C_P* calculation (exp vs. calc).

a – B3LYP/6-31G(d), ***b*** – M06-2X/cc-pVTZ.

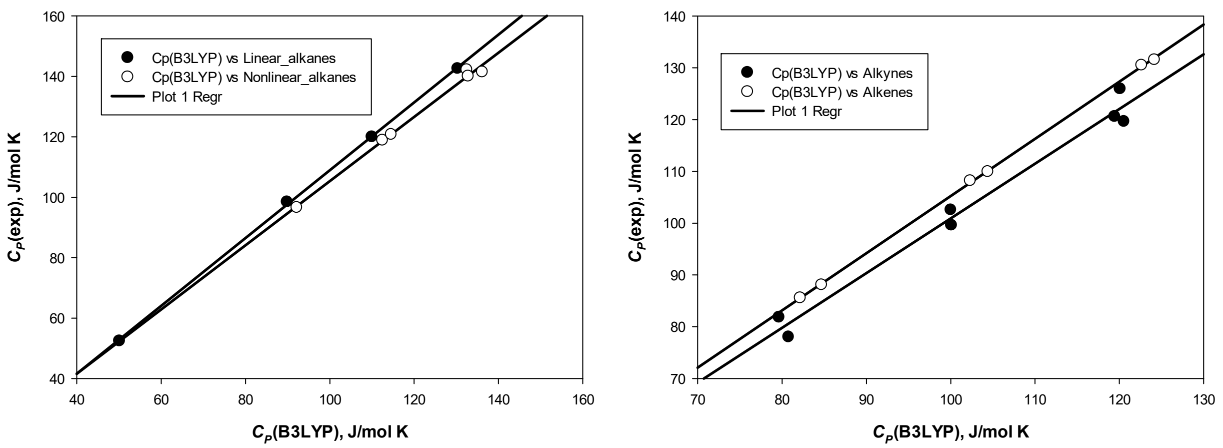

Figure S2. Direct *C_P* calculation (various classes).

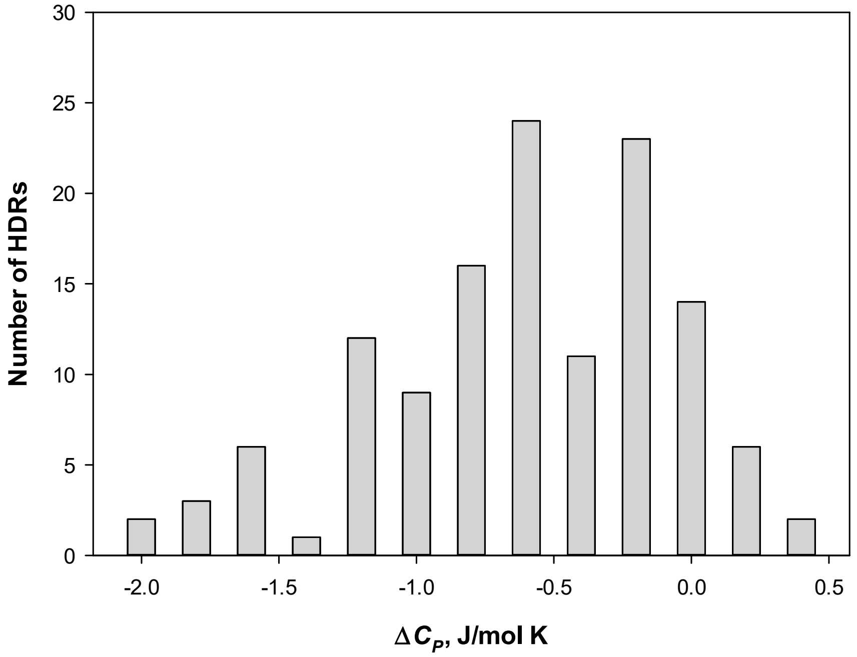

Figure S3. ΔC_P distribution (B3LYP calculations).

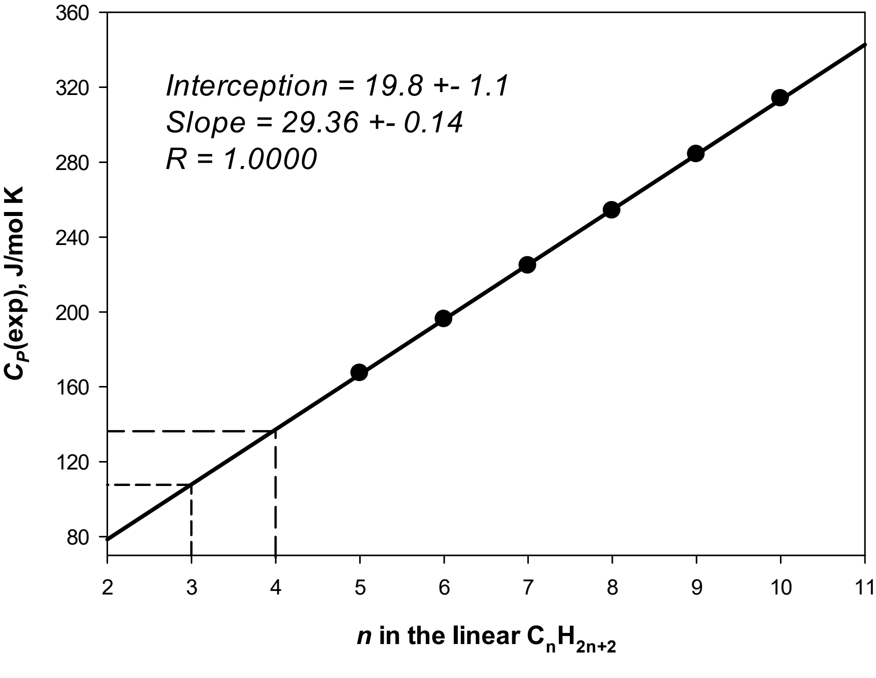

Figure S4. Isobaric molar heat capacities for liquid *n*-alkanes.

Table S3. Coefficients of Shomate equation and calculated absolute enthalpy and heat capacity for linear alkanes

T	T/298.15	C_2H_6	C_3H_8	C_4H_{10}	C_5H_{12}	C_6H_{14}	C_7H_{16}	C_8H_{18}	C_9H_{20}	$C_{10}H_{22}$
200	0.6708	42.30	56.07	76.44	93.55	110.58	127.65	144.77	161.92	179.08
273.15	0.9161	49.46	68.74	92.30	112.55	133.55	154.64	175.69	196.77	217.90
298.15	1.0000	52.49	73.60	98.49	120.00	142.60	165.20	187.80	210.40	233.10
300	1.0062	52.71	73.93	98.95	120.62	143.26	165.98	188.70	211.42	234.18
400	1.3416	65.46	94.01	124.77	152.55	181.54	210.66	239.74	268.82	297.98
500	1.6770	77.94	112.59	148.66	182.59	217.28	252.09	286.81	321.54	356.43
600	2.0124	89.19	128.70	169.28	208.78	248.11	287.44	326.77	366.10	405.85
700	2.3478	99.14	142.67	187.02	231.38	274.05	317.15	360.24	403.34	446.43
800	2.6832	107.94	154.77	202.38	250.62	296.23	342.25	388.28	433.88	479.90
900	3.0186	115.71	165.35	215.73	266.94	315.06	363.59	411.71	459.82	508.36
1000	3.3540	122.55	174.6	227.36	281.58	331.37	381.58	431.37	481.58	531.79
1100	3.6894	128.55	182.67	237.48	293.72	345.18	397.06	448.52	499.99	551.87
1200	4.0248	133.80	189.74	246.27	304.60	357.31	410.45	463.17	516.31	569.44
1300	4.3602	138.39	195.85	253.93	313.80	368.19	422.58	476.98	531.37	585.76
1400	4.6956	142.40	201.21	260.58	322.17	376.56	435.14	489.53	543.92	598.31
1500	5.0310	145.90	205.89	266.40	330.54	389.11	443.50	497.90	556.47	610.86

$C_P = a_0 + a_1\tau + a_2\tau^2 + a_3\tau^3 + a_{-2}\tau^{-2}$, $\tau = T/298.15$, 200 ÷ 1500 K.

	Derived from recommended values					B3LYP/GTBas3	
	a_0	a_1	a_2	a_3	a_{-2}	C_P	H^o
	J/mol·K						Hartree
n-C_nH_{2n+2}							
2	-8.48	64.96	-9.63	0.56	5.10	50.54	-79.759736
3	-16.75	100.42	-16.54	1.07	5.59	70.40	-119.046982
4	-20.03	131.72	-22.23	1.46	7.86	90.73	-158.334240
5	-36.74	173.04	-30.36	2.08	12.22	111.05	-197.621370
6	-50.65	215.53	-40.24	2.93	15.21	131.46	-236.908551
7	-55.29	247.24	-46.06	3.31	16.44	151.72	-276.195617
8	-62.75	281.94	-52.87	3.78	18.28	172.19	-315.482765
9	-76.65	324.34	-62.65	4.61	21.34	192.39	-354.769842
10	-83.61	358.48	-69.17	5.04	23.09	212.84	-394.057032

Table S4. Complete set of HDRs 1 for *n*-alkanes.

$\Delta_r H°$ and ΔC_P were calculated in the B3LYP/GTBas3 level of theory; reaction coefficients of Shomate equation were estimated using data of Table 3S.

Homodesmotic reactions	$\Delta_r H°$	ΔC_P	Δa_0	Δa_1	Δa_2	Δa_3	Δa_{-2}
	kJ/mol	J/mol·K					
**Butane**							
C4H10 + C2H6 --> 2 C3H8	0.03	-0.47	-4.98	4.15	-1.23	0.12	-1.77
**Pentane**							
C5H12 + C2H6 --> C3H8 + C4H10	-0.30	-0.46	8.43	-5.86	1.22	-0.11	-3.87
C5H12 + 2 C2H6 --> 3 C3H8	-0.27	-0.92	3.45	-1.71	-0.01	0.01	-5.65
Average		-0.69	5.94	-3.79	0.61	-0.05	-4.76
StDev		0.33	3.52	2.93	0.87	0.08	1.25
**Hexane**							
C6H14 + C2H6 --> C3H8 + C5H12	-0.17	-0.54	5.64	-7.04	2.96	-0.34	-2.51
C6H14 + C2H6 --> 2 C4H10	-0.51	-0.53	19.06	-17.05	5.41	-0.56	-4.60
C6H14 + 2 C2H6 --> 2 C3H8 + C4H10	-0.48	-1.00	14.07	-12.90	4.18	-0.44	-6.38
C6H14 + 3 C2H6 --> 4 C3H8	-0.44	-1.47	9.09	-8.75	2.95	-0.32	-8.15
Average		-0.89	11.97	-11.43	3.88	-0.42	-5.41
StDev		0.45	5.86	4.48	1.17	0.11	2.42
**Heptane**							
C7H16 + C2H6 --> C3H8 + C6H14	-0.47	-0.40	-3.62	3.75	-1.09	0.13	-0.74
C7H16 + C2H6 --> C4H10 + C5H12	-0.67	-0.47	7.00	-7.44	3.10	-0.32	-1.47
C7H16 + 2 C2H6 --> 2 C3H8 + C5H12	-0.64	-0.94	2.02	-3.29	1.87	-0.21	-3.25
C7H16 + 2 C2H6 --> C3H8 + 2 C4H10	-0.98	-0.93	15.43	-13.30	4.32	-0.43	-5.34

C7H16 + 3 C2H6 --> 3 C3H8 + C4H10	-0.95	-1.40	10.45	-9.15	3.09	-0.31	-7.12
C7H16 + 4 C2H6 --> 5 C3H8	-0.92	-1.87	5.47	-5.00	1.86	-0.20	-8.89
Average		-1.00	6.13	-5.74	2.19	-0.22	-4.47
StDev		0.56	6.61	5.80	1.85	0.19	3.22
**Octane**							
C8H18 + C2H6 --> C3H8 + C7H16	-0.26	-0.61	-0.81	0.75	-0.11	0.04	-1.35
C8H18 + C2H6 --> C4H10 + C6H14	-0.76	-0.54	0.54	0.34	0.03	0.05	-0.32
C8H18 + C2H6 --> 2 C5H12	-0.63	-0.63	-2.25	-0.83	1.77	-0.18	1.05
C8H18 + 2 C2H6 --> C3H8 + C4H10 + C5H12	-0.93	-1.08	6.19	-6.69	2.99	-0.28	-2.82
C8H18 + 2 C2H6 --> 2 C3H8 + C6H14	-0.73	-1.01	-4.44	4.49	-1.20	0.17	-2.09
C8H18 + 2 C2H6 --> 3 C4H10	-1.27	-1.07	19.60	-16.70	5.44	-0.50	-4.92
C8H18 + 3 C2H6 --> 3 C3H8 + C5H12	-0.90	-1.55	1.20	-2.54	1.76	-0.17	-4.59
C8H18 + 3 C2H6 --> 2 C3H8 + 2 C4H10	-1.24	-1.54	14.62	-12.55	4.21	-0.39	-6.69
C8H18 + 4 C2H6 --> 4 C3H8 + C4H10	-1.21	-2.01	9.64	-8.41	2.99	-0.27	-8.47
C8H18 + 5 C2H6 --> 6 C3H8	-1.17	-2.48	4.65	-4.26	1.76	-0.16	-10.24
Average		-1.25	4.89	-4.64	1.96	-0.17	-4.04
StDev		0.64	7.73	6.52	2.04	0.21	3.63
**Nonane**							
C9H20 + C2H6 --> C3H8 + C8H18	-0.44	-0.34	5.63	-6.94	2.86	-0.32	-2.57
C9H20 + C2H6 --> C4H10 + C7H16	-0.73	-0.48	9.80	-10.34	3.98	-0.40	-2.14
C9H20 + C2H6 --> C5H12 + C6H14	-0.90	-0.42	-2.25	-0.73	1.67	-0.16	0.99
C9H20 + 2 C2H6 --> 2 C3H8 + C7H16	-0.70	-0.95	4.82	-6.19	2.76	-0.28	-3.92
C9H20 + 2 C2H6 --> C3H8 + C4H10 + C6H14	-1.21	-0.88	6.18	-6.60	2.89	-0.27	-2.88
C9H20 + 2 C2H6 --> C3H8 + 2 C5H12	-1.07	-0.97	3.39	-7.77	4.63	-0.50	-1.52
C9H20 + 2 C2H6 --> 2 C4H10 + C5H12	-1.41	-0.95	16.80	-17.78	7.08	-0.72	-3.61

C9H20 + 3 C2H6 --> 3 C3H8 + C6H14	-1.17	-1.35	1.20	-2.45	1.66	-0.15	-4.66
C9H20 + 3 C2H6 --> 2 C3H8 + C4H10 + C5H12	-1.38	-1.42	11.82	-13.63	5.85	-0.60	-5.39
C9H20 + 3 C2H6 --> C3H8 + 3 C4H10	-1.71	-1.41	25.23	-23.64	8.30	-0.82	-7.49
C9H20 + 4 C2H6 --> 4 C3H8 + C5H12	-1.34	-1.89	6.84	-9.48	4.63	-0.49	-7.16
C9H20 + 4 C2H6 --> 3 C3H8 + 2 C4H10	-1.68	-1.88	20.25	-19.49	7.08	-0.71	-9.26
C9H20 + 5 C2H6 --> 5 C3H8 + C4H10	-1.65	-2.35	15.27	-15.35	5.85	-0.59	-11.03
C9H20 + 6 C2H6 --> 7 C3H8	-1.62	-2.82	10.29	-11.20	4.62	-0.48	-12.81
Average		-1.29	9.66	-10.83	4.56	-0.46	-5.25
StDev		0.74	7.62	6.53	2.08	0.21	3.88
**Decane**							
C10H22 + C2H6 --> C3H8 + C9H20	-0.15	-0.59	-1.30	1.31	-0.39	0.08	-1.26
C10H22 + C2H6 --> C4H10 + C8H18	-0.62	-0.46	9.31	-9.78	3.70	-0.36	-2.05
C10H22 + C2H6 --> C5H12 + C7H16	-0.57	-0.62	0.07	-3.17	2.37	-0.21	0.47
C10H22 + C2H6 --> 2 C6H12	-0.88	-0.47	-9.20	7.61	-1.68	0.25	2.24
C10H22 + 2 C2H6 --> 2 C3H8 + C8H18	-0.59	-0.93	4.33	-5.63	2.47	-0.24	-3.83
C10H22 + 2 C2H6 --> C3H8 + C4H10 + C7H16	-0.88	-1.08	8.50	-9.03	3.60	-0.32	-3.40
C10H22 + 2 C2H6 --> C3H8 + C5H12 + C6H14	-1.05	-1.02	-3.56	0.57	1.28	-0.08	-0.27
C10H22 + 2 C2H6 --> 2 C4H10 + C6H14	-1.38	-1.00	9.86	-9.44	3.73	-0.30	-2.37
C10H22 + 2 C2H6 --> C4H10 + 2 C5H12	-1.25	-1.09	7.07	-10.61	5.47	-0.54	-1.00
C10H22 + 3 C2H6 --> 3 C3H8 + C7H16	-0.85	-1.54	3.52	-4.88	2.37	-0.20	-5.18
C10H22 + 3 C2H6 --> 2 C3H8 + C4H10 + C6H14	-1.35	-1.47	4.88	-5.29	2.50	-0.19	-4.14
C10H22 + 3 C2H6 --> 2 C3H8 + 2 C5H12	-1.22	-1.56	2.09	-6.46	4.24	-0.42	-2.78
C10H22 + 3 C2H6 --> C3H8 + 2 C4H10 + C5H12	-1.55	-1.55	15.50	-16.47	6.69	-0.64	-4.87
C10H22 + 3 C2H6 --> 4 C4H10	-1.89	-1.54	28.91	-26.48	9.15	-0.86	-6.97
C10H22 + 4 C2H6 --> 4 C3H8 + C6H14	-1.32	-1.94	-0.11	-1.14	1.28	-0.07	-5.92

C10H22 + 4 C2H6 --> 3 C3H8 + C4H10 + C5H12	-1.52	-2.02	10.52	-12.32	5.47	-0.52	-6.65
C10H22 + 4 C2H6 --> 2 C3H8 + 3 C4H10	-1.86	-2.00	23.93	-22.34	7.92	-0.75	-8.75
C10H22 + 5 C2H6 --> 5 C3H8 + C5H12	-1.49	-2.49	5.54	-8.18	4.24	-0.41	-8.42
C10H22 + 5 C2H6 --> 4 C3H8 + 2 C4H10	-1.83	-2.47	18.95	-18.19	6.69	-0.63	-10.52
C10H22 + 6 C2H6 --> 6 C3H8 + C4H10	-1.80	-2.94	13.97	-14.04	5.46	-0.51	-12.29
C10H22 + 7 C2H6 --> 8 C3H8	-1.76	-3.41	8.99	-9.89	4.23	-0.40	-14.07
Average		-1.53	7.70	-8.75	3.85	-0.35	-4.86
StDev		0.81	9.06	8.01	2.64	0.27	4.23

Table S5. Complete set of HDRs 2 for *n*-alkanes.

$\Delta_r H°$ were calculated in the B3LYP/GTBas3 level of theory; coefficients of Shomate equation were corresponded to the alkane.

Homodesmotic reactions	$\Delta_r H°$	a_0	a_1	a_2	a_3	a_{-2}
	kJ/mol	J/mol·K				
**Pentane**						
C5H12 + C3H8 --> 2 C4H10	-0.02	-35.42	173.23	-31.02	2.16	11.27
**Hexane**						
C6H14 + C3H8 --> C4H10 + C5H12	0.07	-45.34	210.47	-38.68	2.74	13.76
C6H14 + 2 C3H8 --> 3 C4H10	0.05	-45.34	210.47	-38.68	2.74	13.76
**Heptane**						
C7H16 + C3H8 --> C4H10 + C6H14	0.09	-55.27	247.71	-46.35	3.33	16.25
C7H16 + C3H8 --> 2 C5H12	0.18	-55.27	247.71	-46.35	3.33	16.25
C7H16 + 2 C3H8 --> 2 C4H10 + C5H12	0.16	-55.27	247.71	-46.35	3.33	16.25
C7H16 + 3 C3H8 --> 4 C4H10	0.14	-55.27	247.71	-46.35	3.33	16.25
**Octane**						
C8H18 + C3H8 --> C4H10 + C7H16	0.16	-65.20	284.95	-54.01	3.91	18.74
C8H18 + C3H8 --> C5H12 + C6H14	0.27	-65.20	284.95	-54.01	3.91	18.74
C8H18 + 2 C3H8 --> 2 C4H10 + C6H14	0.25	-65.20	284.95	-54.01	3.91	18.74
C8H18 + 2 C3H8 --> C4H10 + 2 C5H12	0.34	-65.20	284.95	-54.01	3.91	18.74
C8H18 + 3 C3H8 --> 3 C4H10 + C5H12	0.32	-65.20	284.95	-54.01	3.91	18.74
C8H18 + 4 C3H8 --> 5 C4H10	0.30	-65.20	284.95	-54.01	3.91	18.74
**Nonane**						
C9H20 + C3H8 --> C4H10 + C8H18	0.16	-75.12	322.18	-61.68	4.50	21.23
C9H20 + C3H8 --> C5H12 + C7H16	0.34	-75.12	322.18	-61.68	4.50	21.23

C9H20 + C3H8 --> 2 C6H14	0.36	-75.12	322.18	-61.68	4.50	21.23
C9H20 + 2 C3H8 --> 2 C4H10 + C7H16	0.32	-75.12	322.18	-61.68	4.50	21.23
C9H20 + 2 C3H8 --> C4H10 + C5H12 + C6H14	0.43	-75.12	322.18	-61.68	4.50	21.23
C9H20 + 2 C3H8 --> 3 C5H12	0.52	-75.12	322.18	-61.68	4.49	21.23
C9H20 + 3 C3H8 --> 3 C4H10 + C6H14	0.41	-75.12	322.18	-61.68	4.50	21.23
C9H20 + 3 C3H8 --> 2 C4H10 + 2 C5H12	0.50	-75.12	322.18	-61.68	4.50	21.23
C9H20 + 4 C3H8 --> 4 C4H10 + C5H12	0.48	-75.12	322.18	-61.68	4.50	21.23
C9H20 + 5 C3H8 --> 6 C4H10	0.46	-75.12	322.18	-61.68	4.50	21.23
**Decane**						
C10H22 + C3H8 --> C4H10 + C9H20	0.18	-85.05	359.42	-69.34	5.08	23.72
C10H22 + C3H8 --> C5H12+ C8H18	0.36	-85.05	359.42	-69.34	5.08	23.72
C10H22 + C3H8 --> C6H14 + C7H16	0.45	-85.05	359.42	-69.34	5.08	23.72
C10H22 + 2 C3H8 --> 2 C4H10 + C8H18	0.34	-85.05	359.42	-69.34	5.08	23.72
C10H22 + 2 C3H8 --> C4H10 + C5H12 + C7H16	0.52	-85.05	359.42	-69.34	5.08	23.72
C10H22 + 2 C3H8 --> C4H10 + 2 C6H14	0.54	-85.05	359.42	-69.34	5.08	23.72
C10H22 + 2 C3H8 --> 2 C5H12 + C6H14	0.63	-85.05	359.42	-69.34	5.08	23.72
C10H22 + 3 C3H8 --> 3 C4H10 + C7H16	0.50	-85.05	359.42	-69.34	5.08	23.72
C10H22 + 3 C3H8 --> 2 C4H10 + C5H12 + C6H14	0.61	-85.05	359.42	-69.34	5.08	23.72
C10H22 + 3 C3H8 --> C4H10 + 3 C5H12	0.70	-85.05	359.42	-69.34	5.08	23.72
C10H22 + 4 C3H8 --> 4 C4H10 + C6H14	0.59	-85.05	359.42	-69.34	5.08	23.72
C10H22 + 4 C3H8 --> 3 C4H10 + 2 C5H12	0.68	-85.05	359.42	-69.34	5.08	23.72
C10H22 + 5 C3H8 --> 5 C4H10 + C5H12	0.66	-85.05	359.42	-69.34	5.08	23.72
C10H22 + 6 C3H8 --> 7 C4H10	0.64	-85.05	359.42	-69.34	5.08	23.72

Table S6. Complete set of HDRs 3 for *n*-alkanes.

$\Delta_r H°$ were calculated in the B3LYP/GTBas3 level of theory; coefficients of Shomate equation were corresponded to the alkane.

Homodesmotic reactions	$\Delta_r H°$ kJ/mol	a_0	a_1	a_2	a_3	a_{-2}
		J/mol·K				
**Hexane**						
C6H14 + C4H10 --> 2 C5H12	0.13	-45.01	210.00	-38.44	2.72	13.96
**Heptane**						
C7H16 + C4H10 --> C5H12 + C6H14	-0.17	-55.10	247.47	-46.23	3.32	16.35
C7H16 + 2 C4H10 --> 3 C5H12	-0.03	-55.10	247.47	-46.23	3.32	16.35
**Octane**						
C8H18 + C4H10 --> C5H12 + C7H16	0.05	-65.20	284.95	-54.01	3.91	18.74
C8H18 + C4H10 --> 2 C6H14	-0.25	-65.20	284.95	-54.01	3.91	18.74
C8H18 + 2 C4H10 --> 2 C5H12 + C6H14	-0.12	-65.20	284.95	-54.01	3.91	18.74
C8H18 + 3 C4H10 --> 4 C5H12	0.01	-65.20	284.95	-54.01	3.91	18.74
**Nonane**						
C9H20 + C4H10 --> C5H12 + C8H18	-0.14	-75.29	322.42	-61.80	4.51	21.13
C9H20 + C4H10 --> C6H14 + C7H16	-0.23	-75.29	322.42	-61.80	4.51	21.13
C9H20 + 2 C4H10 --> 2 C5H12 + C7H16	-0.09	-75.29	322.42	-61.80	4.51	21.13
C9H20 + 2 C4H10 --> C5H12 + 2 C6H14	-0.39	-75.29	322.42	-61.80	4.51	21.13
C9H20 + 3 C4H10 --> 3 C5H12 + C6H14	-0.26	-75.29	322.42	-61.80	4.51	21.13
C9H20 + 4 C4H10 --> 5 C5H12	-0.13	-75.29	322.42	-61.80	4.51	21.13
**Decane**						
C10H22 + C4H10 --> C5H12 + C9H20	0.16	-85.39	359.90	-69.59	5.10	23.53
C10H22 + C4H10 --> C6H14+ C8H18	-0.12	-85.39	359.90	-69.59	5.10	23.53

C10H22 + C4H10 --> 2 C7H16	0.10	-85.39	359.90	-69.59	5.10	23.53
C10H22 + 2 C4H10 --> 2 C5H12 + C8H18	0.02	-85.39	359.90	-69.59	5.10	23.53
C10H22 + 2 C4H10 --> C5H12 + C6H14 + C7H16	-0.07	-85.39	359.90	-69.59	5.10	23.53
C10H22 + 2 C4H10 --> 3 C6H14	-0.37	-85.39	359.90	-69.59	5.10	23.53
C10H22 + 3 C4H10 --> 3 C5H12 + C7H16	0.07	-85.39	359.90	-69.59	5.10	23.53
C10H22 + 3 C4H10 --> 2 C5H12 + 2 C6H14	-0.24	-85.39	359.90	-69.59	5.10	23.53
C10H22 + 4 C4H10 --> 4 C5H12 + C6H14	-0.10	-85.39	359.90	-69.59	5.10	23.53
C10H22 + 5 C4H10 --> 6 C5H12	0.03	-85.39	359.90	-69.59	5.10	23.53

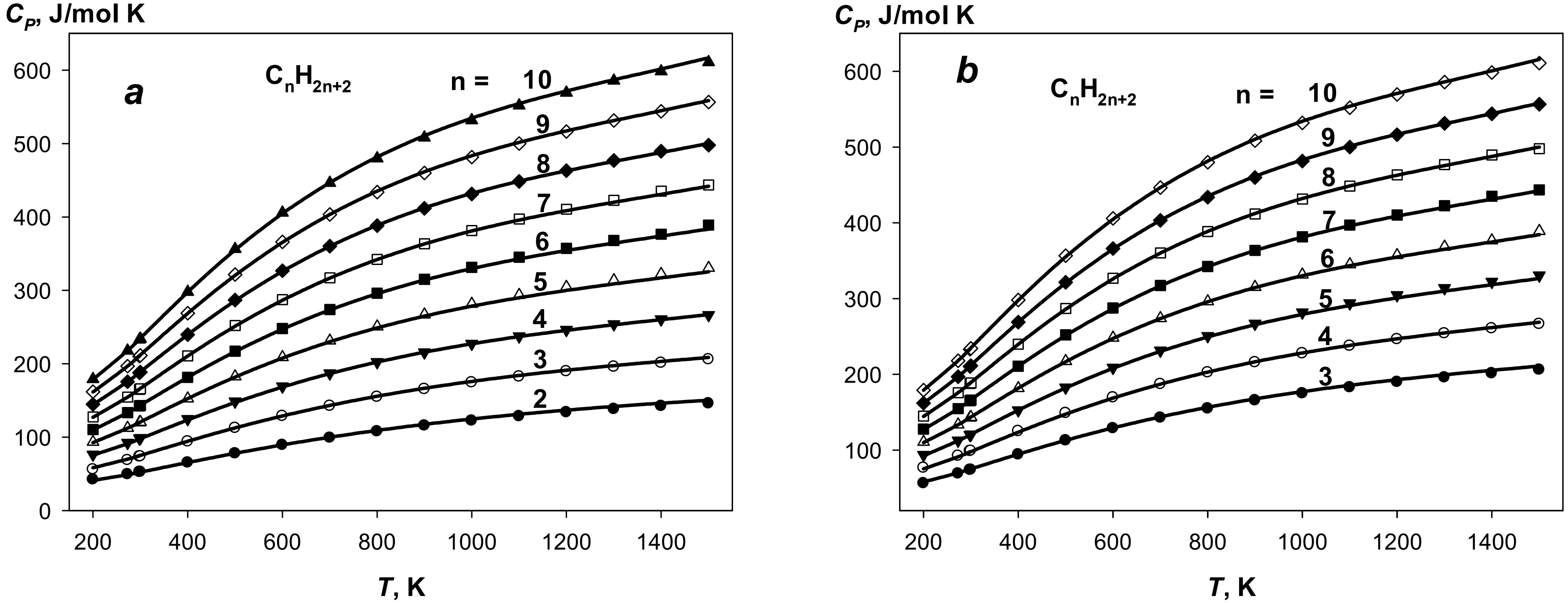

Figure S5. Isobaric heat capacities for *n*-alkanes from HDR 1 (*a*) and 2 (*b*).

The corrected coefficients of Shomate equation from $C_n - C_{10}$ dependence are listed below:

	C_2H_6	C_3H_8	C_4H_{10}	C_5H_{12}	C_6H_{14}	C_7H_{16}	C_8H_{18}	C_9H_{20}	$C_{10}H_{22}$	C_3H_8	C_4H_{10}	C_5H_{12}	C_6H_{14}	C_7H_{16}	C_8H_{18}	C_9H_{20}	$C_{10}H_{22}$
a_0	-6.71	-16.45	-26.19	-35.92	-45.66	-55.40	-65.13	-74.87	-84.61	-15.57	-25.49	-35.42	-45.34	-55.27	-65.20	-75.12	-85.05
a_1	62.82	99.83	136.84	173.84	210.85	247.86	284.87	321.88	358.89	98.76	135.99	173.23	210.47	247.71	284.95	322.18	359.42
a_2	-8.63	-16.19	-23.75	-31.30	-38.86	-46.42	-53.98	-61.53	-69.09	-15.69	-23.35	-31.02	-38.68	-46.35	-54.01	-61.68	-69.34
a_3	0.47	1.04	1.61	2.19	2.76	3.33	3.91	4.48	5.06	0.99	1.58	2.16	2.74	3.33	3.91	4.50	5.08
a_{-2}	4.29	6.69	9.10	11.50	13.90	16.31	18.71	21.12	23.52	6.28	8.78	11.27	13.76	16.25	18.74	21.23	23.72

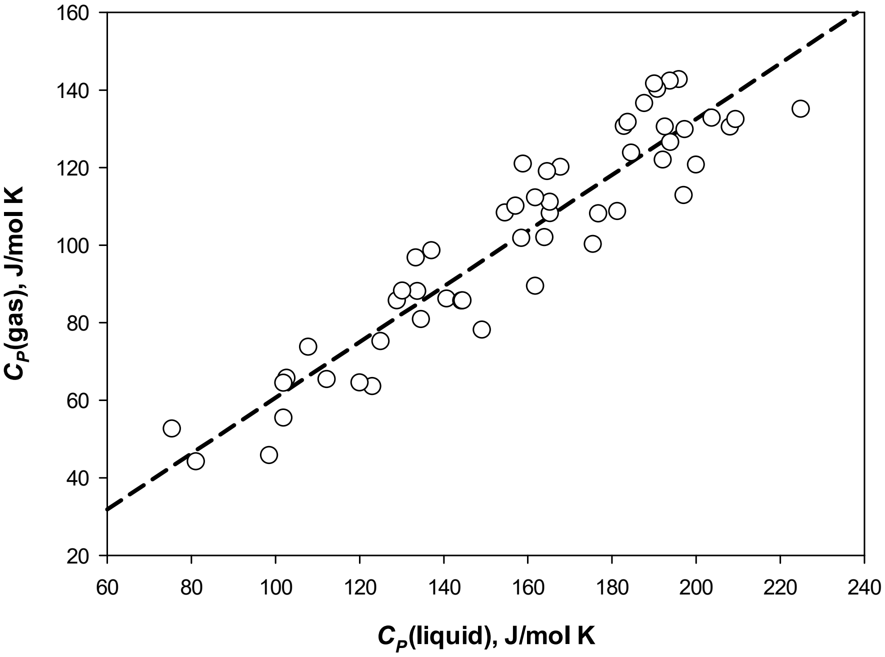

Figure S6. Acree-Chickos correlation of isobaric heat capacities.

C_P(gas) = (0.72 ± 0.04)·C_P(liquid) – (11.2 ± 6.2) J/mol·K, R = 0.94.
